# Deep learning-based analysis of COVID-19 X-ray images: Incorporating clinical significance and assessing misinterpretation

**DOI:** 10.1177/20552076231215915

**Published:** 2023-11-24

**Authors:** Md. Rahad Islam Bhuiyan, Sami Azam, Sidratul Montaha, Risul Islam Jim, Asif Karim, Inam Ullah Khan, Mark Brady, Md. Zahid Hasan, Friso De Boer, Md. Saddam Hossain Mukta

**Affiliations:** 1 Health Informatics Research Laboratory (HIRL), Department of Computer Science and Engineering, 130058 Daffodil International University, Dhaka, Bangladesh; 2 Faculty of Science and Technology, 10095 Charles Darwin University, Casuarina, NT, Australia; 3 Department of Computer Science, 2129 University of Calgary, Calgary, Canada; 4 School of Law, Faculty of Arts and Society, 10095 Charles Darwin University, Casuarina, NT, Australia; 5 Department of Computer Science and Engineering, 130062 United International University (UIU), Dhaka, Bangladesh

**Keywords:** COVID-19, pneumonia, tuberculosis, image processing, Fourier transform, ANOVA test, soft attention layer, misclassified image

## Abstract

COVID-19, pneumonia, and tuberculosis have had a significant effect on recent global health. Since 2019, COVID-19 has been a major factor underlying the increase in respiratory-related terminal illness. Early-stage interpretation and identification of these diseases from X-ray images is essential to aid medical specialists in diagnosis. In this study, (COV-X-net19) a convolutional neural network model is developed and customized with a soft attention mechanism to classify lung diseases into four classes: normal, COVID-19, pneumonia, and tuberculosis using chest X-ray images. Image preprocessing is carried out by adjusting optimal parameters to preprocess the images before undertaking training of the classification models. Moreover, the proposed model is optimized by experimenting with different architectural structures and hyperparameters to further boost performance. The performance of the proposed model is compared with eight state-of-the-art transfer learning models for a comparative evaluation. Results suggest that the COV-X-net19 outperforms other models with a testing accuracy of 95.19%, precision of 96.49% and F1-score of 95.13%. Another novel approach of this study is to find out the probable reason behind image misclassification by analyzing the handcrafted imaging features with statistical evaluation. A statistical analysis known as analysis of variance test is performed, to identify at which point the model can identify a class accurately, and at which point the model cannot identify the class. The potential features responsible for the misclassification are also found. Moreover, Random Forest Feature importance technique and Minimum Redundancy Maximum Relevance technique are also explored. The methods and findings of this study can benefit in the clinical perspective in early detection and enable a better understanding of the cause of misclassification.

## Introduction

Being one of the most widespread reasons for early mortality, lung disease is regarded as a major global health issue. According to the World Health Organization, more than six million people had died as a result of this pandemic worldwide by December 2022.^1^ COVID-19 primarily affects the airways and, as a result, negatively impacts the lungs of affected individuals. It manifests as an upper respiratory tract and lung infection.^2^ Tuberculosis, a recognized communicable disease, is one of the top 10 leading causes of mortality worldwide, and manifests as a chronic pulmonary disease fueled by pathogenic bacterial colonization.^3^ The bacterium known as ‘*Mycobacterium tuberculosis*’ is the primary cause of tuberculosis. Tuberculosis can be successfully resolved with early diagnosis and the subsequent delivery of the appropriate treatment.^4^ Bacterial infection is also a primary factor in the development of pneumonia, a condition marked by inflammation and consolidation of the lung tissue.^5^ By using antimicrobial agents such as antibiotics and antivirals, the manageability of pneumonia can be significantly enhanced. However, prevention of some complications that may result in early mortality, depends on the prompt diagnosis and effective treatment.^6^

Deep learning-based techniques, in particular convolutional neural networks (CNN), have shown remarkable advances in the identification and segmentation of medical images.^7^ Deep learning has made significant strides and these systems are now frequently employed in the research of Computer Aided Diagnosis systems using various medical imaging modalities. Appropriate risk factor prognosis can prevent lung diseases from developing into chronic, serious, and life-threatening problems. This is because prompt diagnosis and careful treatment planning can stop infections from spreading and lung disorders from getting worse, lowering the overall mortality rate. Chest X-ray (CXR) images are considered useful for monitoring and examining several lung conditions, including COVID-19, tuberculosis, and pneumonia. CXR can be used to diagnose lung disorders and research on this topic has shown that these images can provide valuable information about how the condition is evolving.^8^ The CXR patterns of lung disease create differentiation difficulties and frequently result in considerable inter-reader heterogeneity among radiologists.^9^ There is an urgent need for new automated image analysis techniques that can improve radiological qualitative assessments because of probable future waves of diseases and the resulting increase in radiologist workloads. The diagnosis of lung disorders may benefit from a reliable automated process based on deep-learning methods with CXR images. A neural network may concentrate on, and highlight the most crucial areas of, an input image due to the soft-attention process.^10^ In this article, soft attention is integrated in the proposed CNN model to categorize lung disease, drawing inspiration from the work presented by Xu et al.^11^ for image caption generation. Analysis of variance (ANOVA) is arguably the most popular statistical technique for evaluating hypotheses at present. This technique is employed to compare the means of two or more groups that differ significantly from one another.^12^

This study proposes a fully automated and novel deep-learning model to classify CXR images into four classes: normal, COVID-19, pneumonia, and tuberculosis. The images are preprocessed employing several widely used image preprocessing techniques to ensure the highest possible performance of the model. The study also presents an analysis regarding misclassification. Twenty-four handcrafted features are extracted from the images and the difference between the classified and misclassified values for the features are shown by performing ANOVA testing. The automated statistical analysis of misclassification has not previously been undertaken in any notable studies. The major contributions and methods of the study can be summarized as follows:

1. The quality of the images is improved by applying a variety of image preprocessing techniques, including Text Removing, Image Resizing, Morphological Opening, Contrast limited adaptive histogram equalization (contrast limited adaptive histogram equalization [CLAHE]), Histogram Equalization, and Fast Fourier Transform (FFT).
2. A custom CNN model is developed with soft attention mechanism which can interpret lung disorders using X-ray images relevant features prominently and the performance of the proposed model is compared with several transfer learning models, including VGG16, VGG19, InceptionV3, MobileNetV1, MobileNetV2 in terms of accuracy and training time. Additionally, ablation study is carried out for rigorous experiment in order to enhance the accuracy.
3. To identify the differences between classified and misclassified classes an ANOVA test is performed. The ANOVA test is performed twice, firstly, on the four classes concerning each feature, where we find a F-statistics on which point the model can identify a class correctly and another F-statistics at which point the model cannot classify the disease correctly, and secondly, on the individual class concerning each feature which shows highest F-value, and it is the most responsible for the misclassification. Another two-feature selection technique, random forest feature ranking and Minimum Redundancy Maximum Relevance (MRMR) method is used to find out optimal features and justification of ANOVA test results.

## Literature review

Over the past several years, a number of studies have been conducted on the automated detection and classification of lung diseases, including COVID-19. Rahman et al.^3^ performed image preprocessing, data augmentation, image segmentation, and a deep-learning classification approach, to accurately diagnose tuberculosis from CXR images. Nine different transfer learning based deep CNN models were evaluated. The best-performing model, ChexNet, had accuracy, precision, sensitivity, F1-score, and specificity of 96.47%, 96.62%, 96.47%, 96.47%, and 96.51%, respectively. Ahn et al.^13^ introduced a new technique that integrates sparse spatial pyramid (SSP) features from a local image dictionary with domain-transferred CNNs (DT-CNNs) to increase the performance of X-ray image categorization. Very deep CNNs provided by the VGG have been used. The DT-CNNs and SSP successfully identified the classes with 47% and 82% recall, respectively. Pereira et al.^14^ proposed a classification scheme based on the following viewpoints: (i) a classification with multiple classes; (ii) a hierarchical classification. Over-sampling and under-sampling methods were applied to balance the dataset. The proposed approach tested in RYDLS-20 produced a macro-average F1-score of 0.65 and an F1-score of 0.89 for the COVID-19 identification in the scenario of hierarchical classification. Munusamy et al.^15^ developed a novel FractalCovNet architecture utilizing U-Net and Fractal blocks to split chest CT scan pictures and locate the lesion sites. Transfer learning was also utilized to classify CXR images using the same FractalCovNet architecture, and the classification result was compared. FractalCovNet got an accuracy of 99% where the precision, recall, and F1 score was 99%, 87%, and 92%, respectively. Hassanien et al.^16^ proposed a learning-based method for the detection of COVID-19-infected patients using X-ray images. The multilevel thresholding with the support vector machine (SVM) approach was proposed, which demonstrated superior classification performance with COVID-19 for infected lung identification. According to the findings of the suggested model, the lung classification had an average sensitivity, specificity, and accuracy of 95.76%, 99.7%, and 97.48%, respectively. Abbas et al. 2020^17^ developed a model labeled Decompose, Transfer, and Compose (DeTraC), to classify COVID-19 using CXR images. DeTraC was successful in identifying COVID-19 X-ray pictures from other cases with moderate acute respiratory syndrome and cases with severe acute respiratory syndrome with a high accuracy of 93.1%. Guan et al.^18^ considered the issue of multilabel thorax disease classification on CXR images and proposed a model which is a category-wise residual attention learning (CRAL) framework. A class-specific attentive view predicted the existence of numerous diseases using CRAL. The average area under the receiver operating characteristics curve (AUC) score obtained by CRAL was 81%. Mueen et al.^19^ proposed a content-based image retrieval method which used multilevel image features and a state-of-the-art machine learning method, SVM. They extracted three levels of features: global, local, and pixel and combined them in one large feature vector and it achieved a recognition rate of 89%. Sharma et al.^20^ introduced deep CNN architectures to extract features from CXR images and classify pneumonia with both augmented and original datasets. Among the models, model 1 (with an augmented dataset and dropout layer) achieved the highest testing accuracy of 90.68%. Avuçlu^21^ experimented with five different machine learning algorithms, Multi-Class SVM, k Nearest Neighbor, Decision Tree, Multinomial Logistic Regression, Naive Bayes (NB), where NB performed best in terms of testing accuracy (93.87%). In addition, the Cohen Kappa test was used to determine the dependability of agreement between two or more observers. Heidari et al.,^22^ proposed a transfer learning-based CNN model based on VGG16 where the CNN model yielded 94.5% accuracy. In the study, the original images were processed to create two sets of filtered images using a bilateral filter and a histogram equalization technique. The CNN deep-learning model’s three input channels are then fed with the original image and the two filtered images, increasing the model’s capacity for learning. Hussain et al.,^23^ proposed a deep CNN model called CoroDet for the classification of COVID-19, and for four-class classification, the model achieved an accuracy of 91.2%. In Table 1, the methodology, findings and results and limitation of the papers are pointed out.

**Table 1.** Summarizing table of literature review.

Authors	Year	Methodology	Findings and result	Limitation
Rahman et al.^3^	2020	Image preprocessing, data augmentation, image segmentation, and classification using transfer learning-based model	Highest test accuracy 96.47%,	Binary classification, inadequate image preprocessing
Ahn et al.^13^	2016	Integration of SSP features from a local image dictionary with DT-CNNs using VGG	Highest recall 82%	Image preprocessing approaches have undergone fewer experiments
Pereira et al.^14^	2020	Multiclass and hierarchical classification, utilizing over-sampling and under-sampling methods to balance the dataset	Highest F1-score 89%	No evaluation of the suggested model's performance in relation to other models
Munusamy et al.^15^	2021	Development of FractalCovNet architecture using U-Net and Fractal blocks for lesion site localization in chest CT scan images. Transfer learning is used for classification	Highest test accuracy 99%	Insufficient image preprocessing
Hassanien et al.^16^	2020	Learning-based method, multilevel thresholding with SVM approach for infected lung identification	Highest test accuracy 97.48%	Fewer experiments with image-processing techniques and a limited number of classification models
Abbas et al.^17^	2020	Model labeled Decompose, Transfer, and Compose (DeTraC) for classifying COVID-19 using CXR images	Highest test accuracy 93.1%	Confined dataset, insufficient hyperparameter tweaking
Guan and Huang^18^	2018	CRAL framework for multilabel thorax disease classification on CXR images	Average AUC score 81%	Insufficient image processing
Mueen et al.^19^	2007	CBIR method using multilevel image features and SVM	CBIR method achieved a recognition rate of 89%	Lack of performance comparisons between the suggested model and other models
Sharma et al.^20^	2020	Deep CNN architectures for feature extraction and classification of pneumonia in CXR images using augmented and original datasets	Highest test accuracy 90.68%	Fewer investigations on imagine preprocessing techniques, and inadequate model robustness analysis
Avuçlu^21^	2022	Experimentation with five machine learning algorithms for COVID-19 classification using CXR images	Highest test accuracy 93.87%	Minimal experimentation with image processing methods, insufficient dataset
Heidari et al.^22^	2020	VGG16 with bilateral filtering and histogram equalization technique	Highest test accuracy 94.5%	Lack of an evaluation of the efficacy of the suggested model against different models
Hussain et al.^23^	2021	Deep CNN model called CoroDet for the classification of COVID-19	Highest test accuracy 91.2%	Small dataset, inadequate hyperparameter tuning, lack of performance evaluations between the proposed model and other models
Al-Shourbaji et al.^24^	2022	A model called BNCNN was introduced, data preprocessing, feature extraction, and classification	Highest test accuracy 99.14%	Insufficient investigations to determine the underlying causes of the misinterpretation
Anter et al.^25^	2021	Proposed a new model called AFCM-LSMA uses adaptive Fuzzy C-means and an improved SMA for diagnosing COVID-19	Highest test accuracy 96%	Lack of analysis to ascertain the causes of misclassification
Proposed study		This method is proposed for automated classification of CXR image using a novel COV-X-net19 model with effective image preprocessing and finding out the reason of misinterpretation of misclassified image	COV-X-net19 model achieves 95.19% test accuracy	Optimization of proposed model can be done by modifying the scratch implementation of the CNN layers in order to increasing the accuracy

AFCM-LSMA: adaptive fuzzy C-means with level set method and affine transformation; AAUC: The average area under the receiver operating characteristics curve; BNCNN: binary neural network with convolutional neural network; CBIR: content-based image retrieval; CNN: convolutional neural network; CRAL: category-wise residual attention learning; CT: computed tomography; CXR: chest X-ray; DT: domain-transferred; SMA: slime mould algorithm; SSP: sparse spatial pyramid; SVM: support vector machine; VGG: visual geometry group.

The outcome of the research is to classify CXR images into four categories: normal, COVID-19, pneumonia, and tuberculosis with automated and innovative process. To achieve the best potential performance of the model, images are preprocessed using a variety of widely used image preprocessing techniques. A misclassification analysis is also presented. Twenty-four manually extracted features are taken from the images, and an ANOVA test is used to demonstrate the difference between correctly and incorrectly classified values for each feature. Moreover, random forest feature selection and MRMR methods implemented for finding out the optimal features. From the previous study presented in Table 1, no prominent studies using automated statistical analysis of misclassification have been conducted before. Moreover, effective image preprocessing techniques with optimal parameter values and developing a CNN model with ablation study is another novel approach of the study.

## Methodology

In recent years, there has been a growing interest in utilizing deep-learning techniques to improve the accuracy and efficiency of medical image analysis. One particular application is the classification of CXR images, which is a critical task in effectively diagnosing various respiratory diseases. In this study, we introduce a deep-learning approach to classify CXR images into four classes, with the aim of improving diagnostic accuracy and reducing the workload of medical professionals.

**Figure 1.**
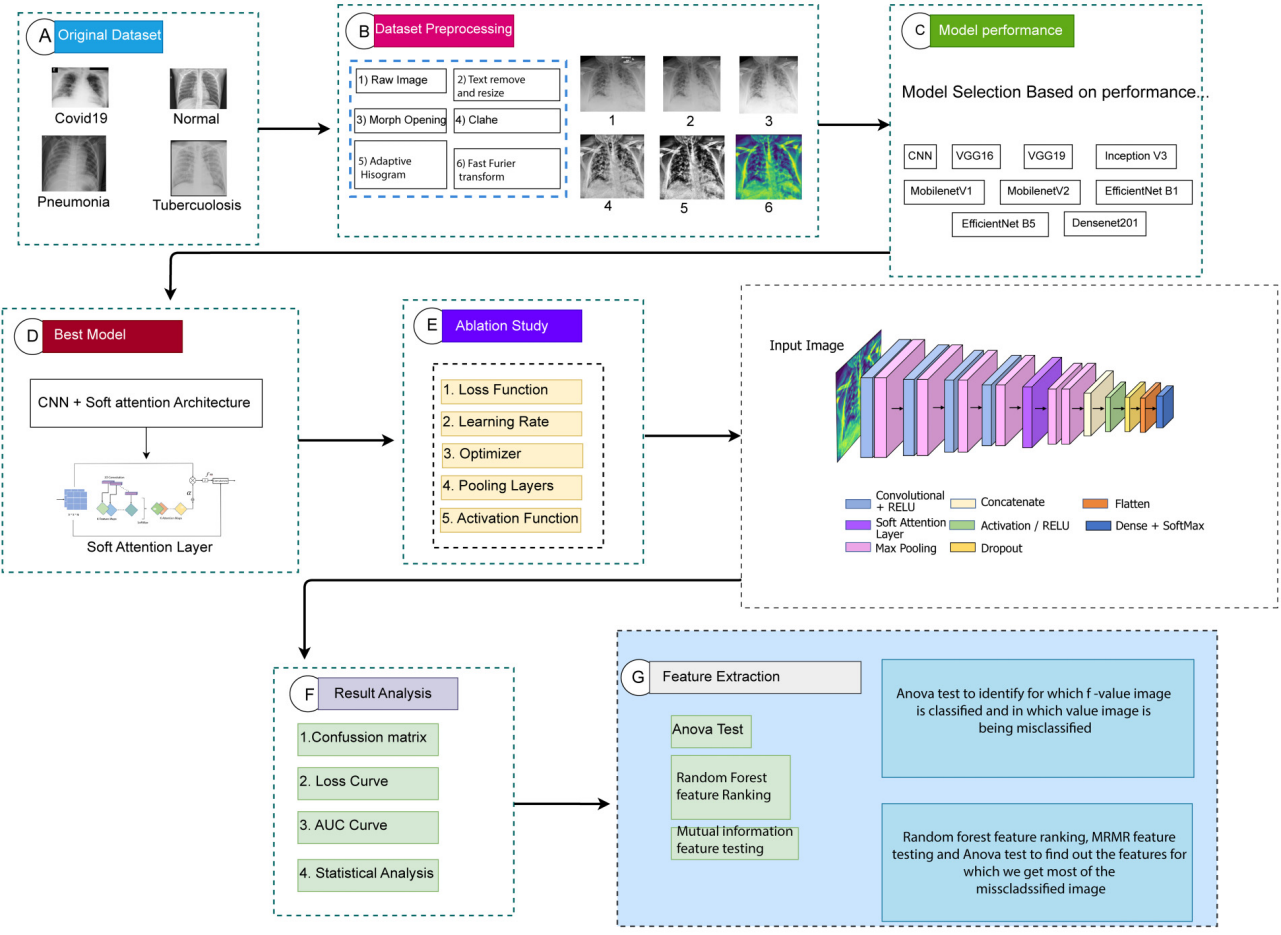
Demonstration of the proposed methodology.

The process and results of our study, including the data preprocessing, the deep-learning model with an additional soft attention layer, feature extraction, and statistical analysis are presented in Figure 1.

The entire workflow of the study can be summarized as follows:

1. The dataset analyzed in this study is imbalanced, consisting of 7106 images consisting of four classes which are COVID-19, normal, pneumonia, and tuberculosis, respectively.
2. Images in the dataset are found with uneven size, noise, and visible artifacts. To address these challenges and to achieve a high level of accuracy, a number of image preprocessing approaches are employed, including text removal, image resizing, morphological opening, CLAHE, histogram equalization, and FFT.
3. A deep-learning model is developed based on deep CNN with an additional soft attention layer to perform the classification. Though our dataset is unbalanced, this model is able to achieve optimal performance.
4. In order to achieve the maximum performance from our proposed model, an ablation study is applied. The activation function, pooling layers, learning rate, optimizer, and loss function are the backbone of the ablation study.
5. The performance of the model is compared with eight transfer learning models, named VGG16, VGG19, MobileNetV2, MobileNet, Inception V3, Densenet201, EfficientNetB1, and EfficientNetB5.
6. Feature analysis is a novel part of this study used in finding the features that are responsible for both correctly classifying and misclassifying an image. Twenty-four features are extracted from the region of interest (ROI) of classified and misclassified images with the goal of determining the factors of misclassification. We next separate the values into two autonomous data frames to obtain improved statistical information. Finding the features that led to the misclassification is the main objective of this experiment. In order to assess the importance and influence of each characteristic on the probability of misclassifications, we conducted an ANOVA test using the features. The discriminating potential of individual characteristics and their impact on classification accuracy are both insightfully examined in this investigation. It should be emphasized that while the CNN and transfer learning models’ roles were only focused on classification, the handcrafted features are solely used in ANOVA test, Random Forest feature selection and MRMR method.
7. The ANOVA test is used to find out the difference between the features of a particular class. In the ANOVA test, an F-value indicates how distinct two different classes are. The test is applied with four classes at a time and the feature is found for which our proposed model can classify the class accurately and finds the values for which our model is not able to classify an image.
8. In a second analysis, the ANOVA test is performed between the features of classified and misclassified images, and some features are found to differ significantly from those of a classified image. This suggests that features with the highest F-value in the ANOVA test are therefore responsible for incorrect image classification. In addition, random forest feature ranking and MRMR method are applied to the extracted features to find out the optimal features and to justify the ANOVA test results.

## Dataset

**Figure 2.**
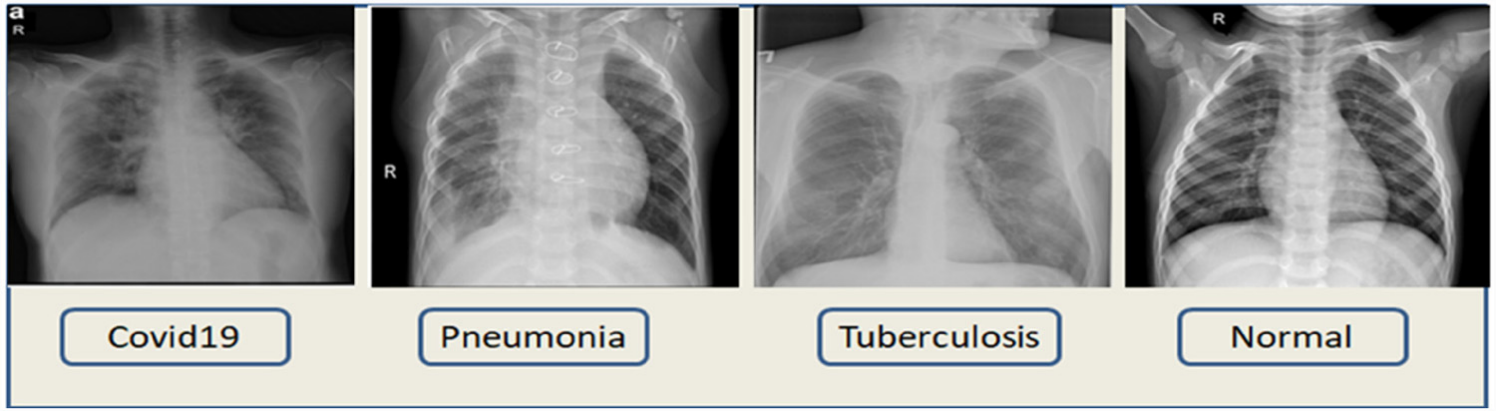
Images of the four classes of the dataset.

The dataset^26^ used in this study is a merged dataset of four publicly available sources with a total of 7135 images. Four distinct classes are obtained: COVID-19, normal, pneumonia, and tuberculosis classes. The authors of this dataset declared that pneumonia and normal images were selected from retrospective cohorts of one to 5-year-old pediatric patients at the Guangzhou Women and Children's Medical Center.^27^ All CXR imaging was done as part of the regular clinical treatment provided to patients. All chest radiographs of pneumonia and normal images were initially checked for quality control before being removed from the study of the CXR pictures. The images were graded by two qualified physicians before the diagnosis and the image could be used to train the artificial intelligence system. A third expert also reviewed the evaluation set to make sure there were no grading mistakes. The tuberculosis images were collected from the National Institute of Allergy and Infection Disease tuberculosis (TB) portal.^28^ A database of chest X-ray images for TB positive cases as well as normal images has been created by a team of researchers from Qatar University, Doha, Qatar, and the University of Dhaka, Bangladesh, along with their collaborators from Malaysia and Hamad Medical Corporation. Currently, there are 700 TB of publicly available images in their release. Reverse transcription polymerase chain reaction (RT-PCR) is used for diagnosis of the COVID-19. The X-ray machines are widely available and provide images for diagnosis quickly so CXR images can be very useful in early diagnosis of COVID-19.^29^ RT-PCR images were collected from this particular source. Also, some images are collected from a GitHub source, which is approved by the University of Montreal's Ethics Committee #CERSES-20-058-D.^30^ In Table 2 the number of images along with class is described and Figure 2 represents the images of each class.

**Table 2.** Dataset description.

Name	Description
Total number of image	7135
Color grading	Red Green Blue (RGB)576
COVID-19	574
Normal	1583
Tuberculosis	703
Pneumonia	4273

It can be observed from Table 2 that out of 7135 total images, the pneumonia class contains more than half of the images of 4273 and normal class contains 1583 images. The rest two classes comprise less than 100 images. Therefore, this dataset can be considered as an imbalanced dataset from where obtaining optimal classification accuracy might be challenging. In Figure 2, the images of four classes are shown.

As the dataset was generated by merging four datasets of different sources, variations based on intensity level, image quality and pixel size may exist among the images which might cause poor performance of the model. In order to deal with and overcomes these variations, ablation study and several image preprocessing techniques have been introduced. In addition, the parameters of the image preprocessing algorithms are determined through extensive experiments in order to achieve the best outcome even with the variations among the images. The aim is to enhance the image quality without losing the necessary information. In ablation study, the model is optimized by experimenting with different layer configurations and hyperparameters.

## Image preprocessing

One of the key steps to ensure that the model performance and computation times are both optimal image preprocessing, which is undertaken before feeding the images into the neural network.^31^ This study applies several widely used methods to perform artifact removal and image quality improvement. The CXR pictures of this study contain several artifacts, noise, and poor contrast. Morphological opening^32^ is applied for the removal of artifacts from the images. The images are then enhanced with CLAHE^33^ to adjust their contrast and brightness. Histogram Equalization^34^ is then applied to enhance the contrast of the images even further. In order to filter out noise, FFT^35^ is lastly used.

### Text removal

Text removal is useful for a variety of purposes and is necessary for effectively identifying and segmenting the significant region of a CXR image. This method uses Optical Character Recognition to find text hidden within images and inpainting, which fills in the blanks in parts of an image to create a whole image. Firstly, the method applies a mask to each bounding box to define the algorithm that which part of the image should be inpainted. The text in the image is then identified and the bounding box coordinates of each text is then determined and finally using an *inpainting* algorithm the masked areas of the image are inpainted, resulting in a text-free image.^36^

### Morphological opening

In this study, morphological opening is applied as a preprocessing step to satisfy our main goal of eliminating small, undesired information or noise from the image. Both morphological opening and closing operations can be applied in removing particular types of artifacts, depending on the characteristics of the artifacts and the expected outcome. Morphological opening is usually preferred for artifact removal and noise reduction, due to its efficiency in eliminating small, isolated artifacts. The algorithm is more effective since it preserves object boundaries and prevents the possible merger of nearby items. Morphological closing is more suitable in filling gaps or holes in objects or merging fragmented structures. Using binary thresholding, the image is first transformed into a binary representation before morphological opening is applied.^32^ Using a kernel, the morphological opening is applied to the binary image. Based on the properties of the artifacts to be removed, the size and shape of the kernel are chosen. The process starts with an erosion step in which a structural element is scanned over the image. The regions or objects in the image are effectively eroded or shrunk during this erosion process. The dilation phase is performed after the erosion process. Morphological opening removes small, isolated, and bright parts while keeping the larger features in the image by sequentially applying erosion and dilation. The smaller portions or artifacts are eliminated during the erosion step, and the remaining regions are then resized during the following dilation step. A smoother image with less noise and isolated artifacts is the result. The opening of an image *f* by a structuring element *s*, denoted by (fθs) , is an erosion followed by a dilation. This can be represented as equation (1).

(1) fos=(fθs)⊕s

**Figure 3.**
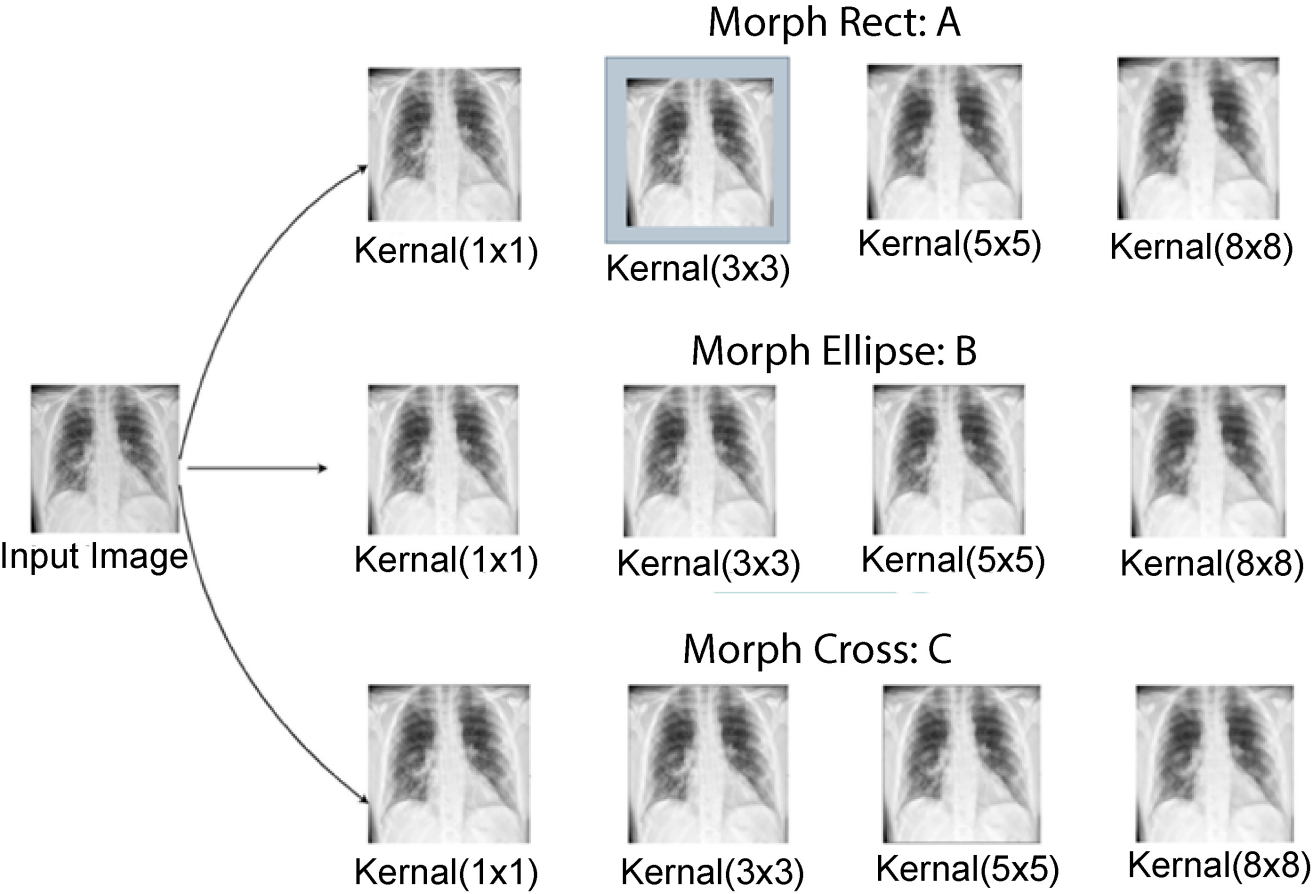
Outputs of several parameters testing (a) morph rect parameter testing, (b) morph ellipse parameter testing, and (c) morph cross parameter testing.

Four kernel sizes are experimented with including 1  ×  1, 3  ×  3, 5  ×  5, and 8  ×  8. For a smaller kernel size of 1  ×  1, the artifacts are not removed properly. On the other side, applying a larger kernel such as 8  ×  8 causes loss of important information. Hence, 3  ×  3 kernel size is selected as an appropriate size for this particular task. In addition, three kernel shapes are also experimented with morph rect, morph ellipse, and morph cross. The identical structural element is scanned over the degraded image, and the center pixel is set to white if at least one pixel in the neighborhood it covers is white. It is set to black if not. The regions or objects in the image are expanded or distorted during this dilation step, partially regaining their former size and shape. In Figure 3, the outputs have been shown after experimenting with several parameter settings.

After experimenting with different kernel shapes and sizes, a rectangular kernel of size 3  ×  3 with kernel shape morph rect is used since it successfully removes artifacts while maintaining the relevant information.

### CLAHE

**Figure 4.**
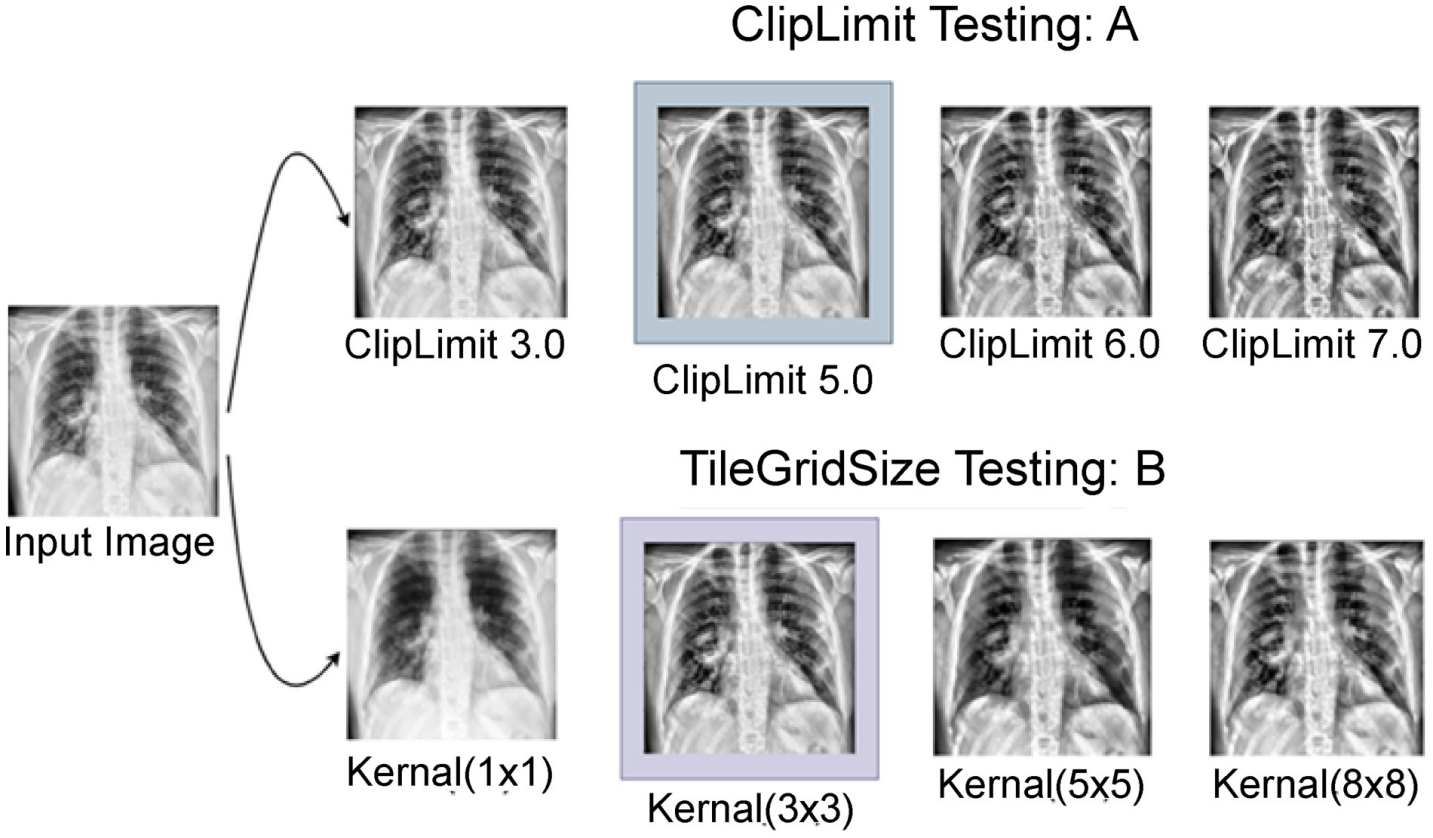
CLAHE parameter meter testing (a: ClipLimit testing and b: TileGridSize testing). CLAHE: Contrast limited adaptive histogram equalization.

By correcting over-amplification of contrast levels, CLAHE is used to balance the overall contrast. Instead of working with the entire image, the algorithm splits it into little areas called tiles and performs operations on each tile.^37,38^ Two parameters are required to apply CLAHE: cliplimit *and* tilegrid size*.* For each tile, the maximum permitted contrast enhancement is set by the “cliplimit” parameter. Higher “cliplimit” values enable more contrast enhancement, but they also increase visibility of noise. This experiment explores various “cliplimit” values between 1 and 5. Additionally, we have also changed the tile grid's dimensions. The number of tiles used to divide the image depends on the size of the tile grid. After experimenting with several parameter settings of cliplimit and tilegrid size, the optimal configuration is determined. This combination most likely achieved an excellent balance between increasing contrast and maintaining the image's details and information. In Figure 4, the outputs have been shown after experimenting with several parameters.

After several tests on our dataset with various parameter values, the optimum ClipLimit and TileGridSize sizes were determined to be 5.0 and 3  ×  3.

### Histogram Equalization

Histogram Equalization is a technique used to enhance the contrast of an image by redistributing the pixel intensities. Its autonomous operation and effective presentation of all contrast available in the image data have been demonstrated in medical imaging.^31^ The algorithm works by mapping the original intensity values to new values that spread out over the entire dynamic range of the image.

### Fast Fourier transform

The Fourier transform is frequently referred to as a generalization of the Fourier series. The Fourier transform helps to extend the Fourier series to nonperiodic functions, and any function can be regarded as a collection of simple sinusoids.^32^ The function of the Fourier transform is given below in equation (2):

(2) $$f(x)=\int_{-\infty }^{\infty }\;F(k)e^{2\pi ikx\;}dk$$

**Figure 5.**
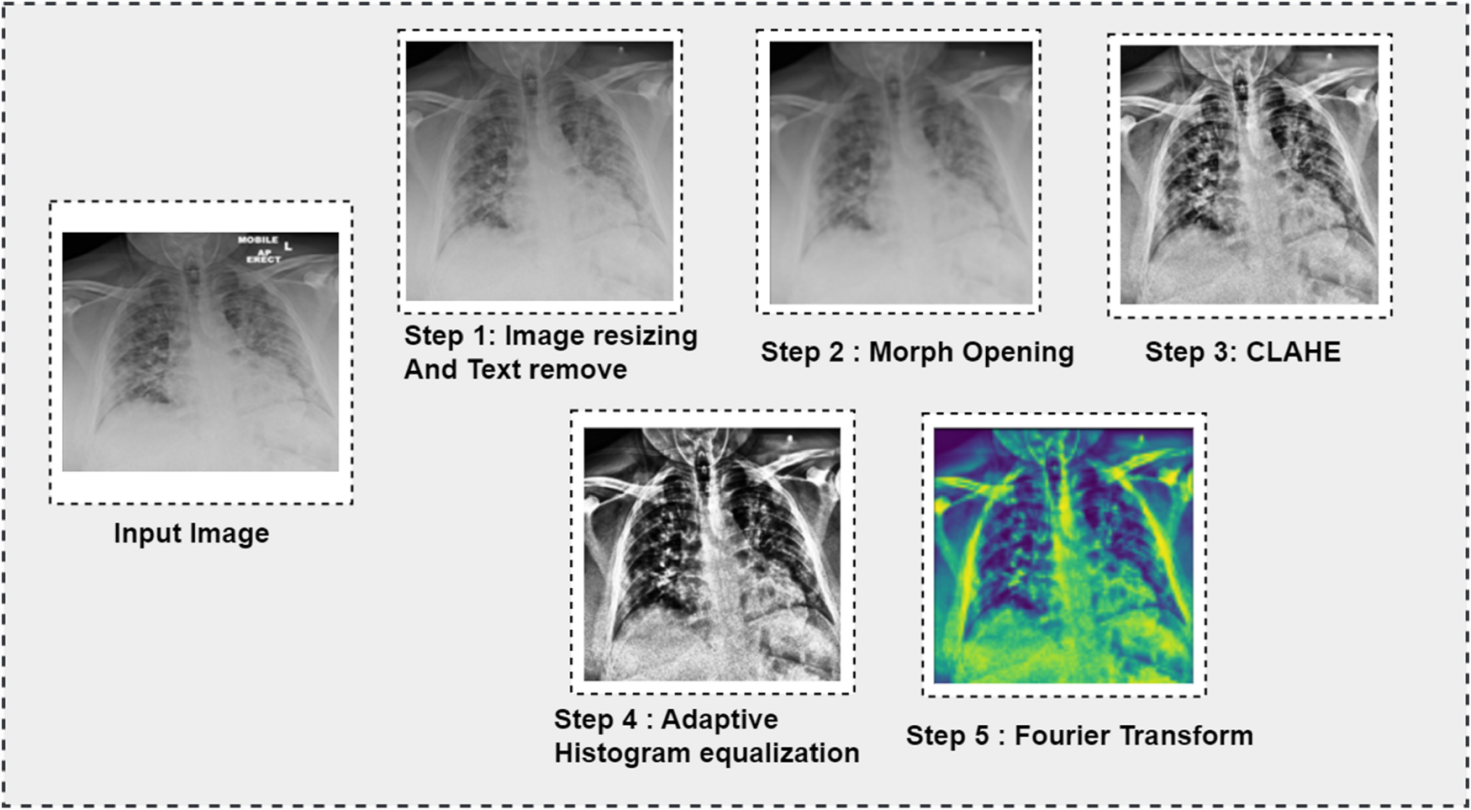
Image preprocessing steps with outputs.

**Figure 6.**
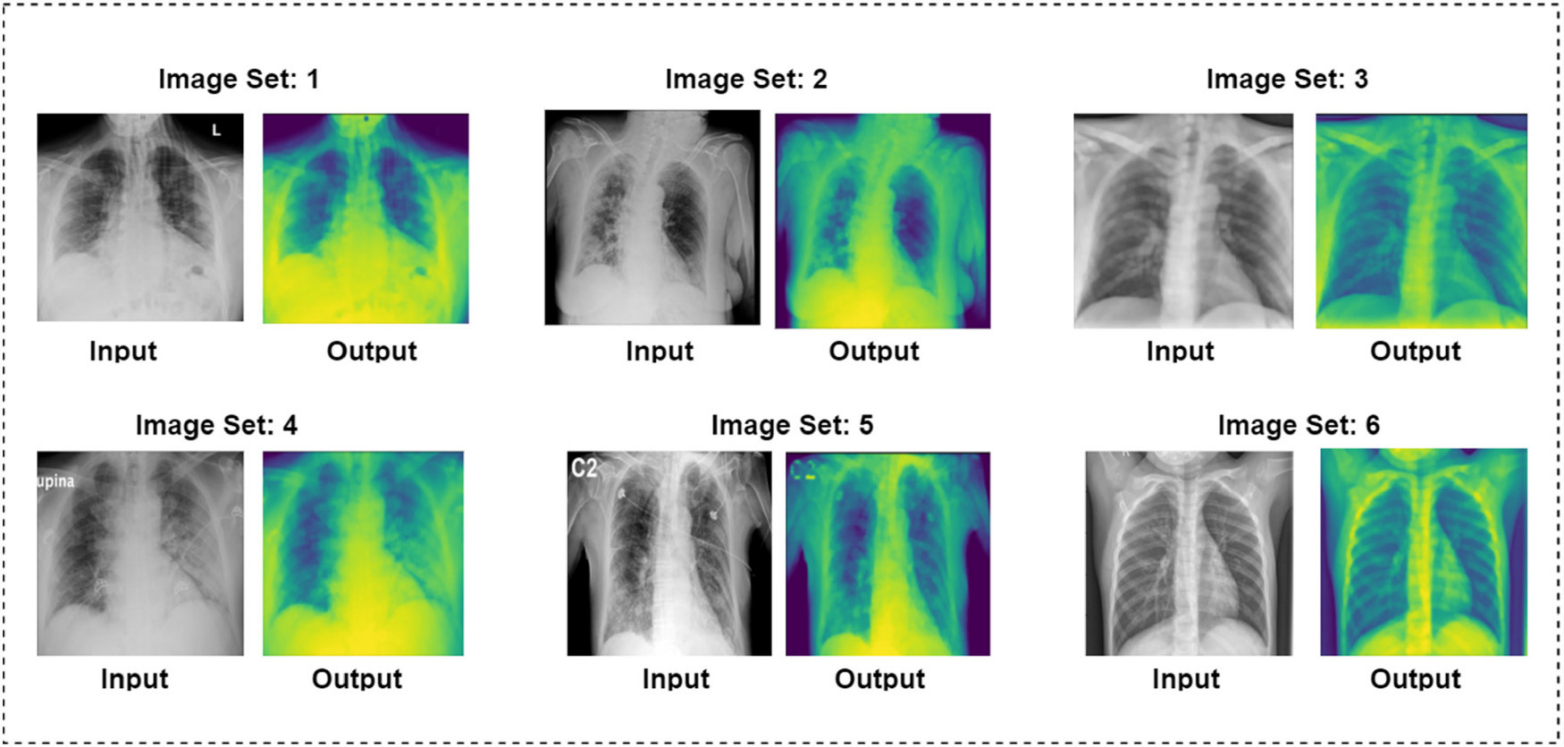
Six sets of preprocessed images where input and output images are shown.

The whole image preprocessing procedure, from the removal of artifacts through image enhancement, is shown below in Figure 5, and Figure 6 demonstrates six sets of preprocessed images where both input and output images have been shown.

## Model evaluation

### Dataset split

To train the proposed model and other transfer learning models, the dataset is split into train, validation, and test sets with a ratio of 70:10:20, respectively. Table 3 represents the number of images in training, validation, and test set across each class after splitting.

**Table 3.** Number of images in each class after splitting.

Disease name	Number of images	Train	Test	Validation
COVID-19	576	403	115	58
Pneumonia	4273	2991	855	427
Tuberculosis	703	492	140	71
Normal	1583	1108	316	159
Subtotal	∑7135	∑4994	∑1426	∑715

### Base model architecture

At first a CNN baseline CNN model is developed which is improved with an ablation study and thus the proposed model architecture is obtained. The performance of the model is later compared with eight transfer learning models. CNN is a type of deep-learning neural network architecture commonly used in image and video analysis tasks, such as image classification, object detection, and segmentation. It is composed of several layers, including convolutional layers, pooling layers, and fully connected layers. The convolutional layers in a CNN are used to extract relevant features from the input image. Each convolutional layer consists of a set of learnable filters that convolve with the input image to produce a set of output feature maps. The filters are typically small in size and are designed to detect specific features such as edges, corners, and textures. The pooling layers in a CNN are used to down sample the output feature maps produced by the convolutional layers. This helps to reduce the spatial dimensionality of the input image and control overfitting. The most commonly used pooling operation is max-pooling, which takes the maximum value within a small subregion of the input. The fully connected layers in a CNN are used to classify the input image based on the features learned by the convolutional layers. These layers are similar to those found in a traditional feedforward neural network and connect all neurons in one layer to all neurons in the next layer. Through the use of appropriate training data, CNNs are able to learn data-driven, highly representative, layered hierarchical image characteristics.^9^

The base model consists of 15 layers: 4 convolutional, 6 max-pooling, 1 dropout layer with a dropout factor of 0.5. A flatten layer is applied before the last dense classification layer, and a final dense layer for classification. A 3  ×  3 kernel size is used for the convolution layers, and nonlinearity is introduced by including the Rectified Linear Unit (ReLU) activation function. The first four max-pooling layers are followed by one convolutional respectively. After adding eight layers (4 convolutional and 4 max-pool), 2 max-pool layers are added. Then two max-pooling layers are added followed by a concatenation layer to concatenate the outputs of the previous max-pooling layers. The classification is performed through a SoftMax activation function.

### Proposed model (COV-X-net19)

**Figure 7.**
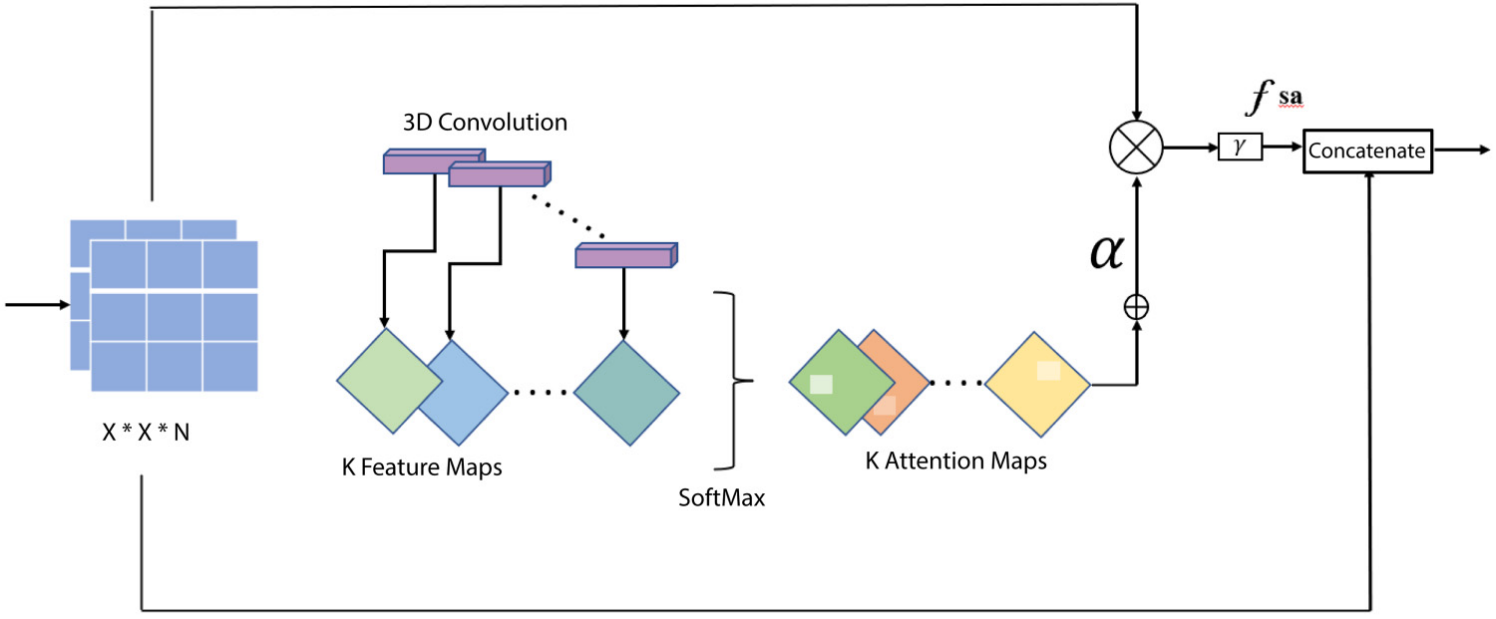
Soft attention mechanism.

Deep-learning models need a lot of processing power and training time; therefore, we work to keep the model architecture as simple as possible to cut down both factors. The proposed CNN model is optimized through an ablation study on base CNN architecture where an additional soft attention layer is introduced. The attention mechanism enables the network to concentrate on various regions of the input image while accounting for the varied significance of various regions in the final prediction. Each component of the input image receives a weight from the attention layer, indicating how significant it is to the final prediction. The features that are collected from the image are then combined using the weights to form a weighted sum, which is utilized as input to the network's next layer. This enhances performance by enabling the model to concentrate on the most important image elements.^10^ The architecture of the soft attention mechanism is given in Figure 7.

**Figure 8.**
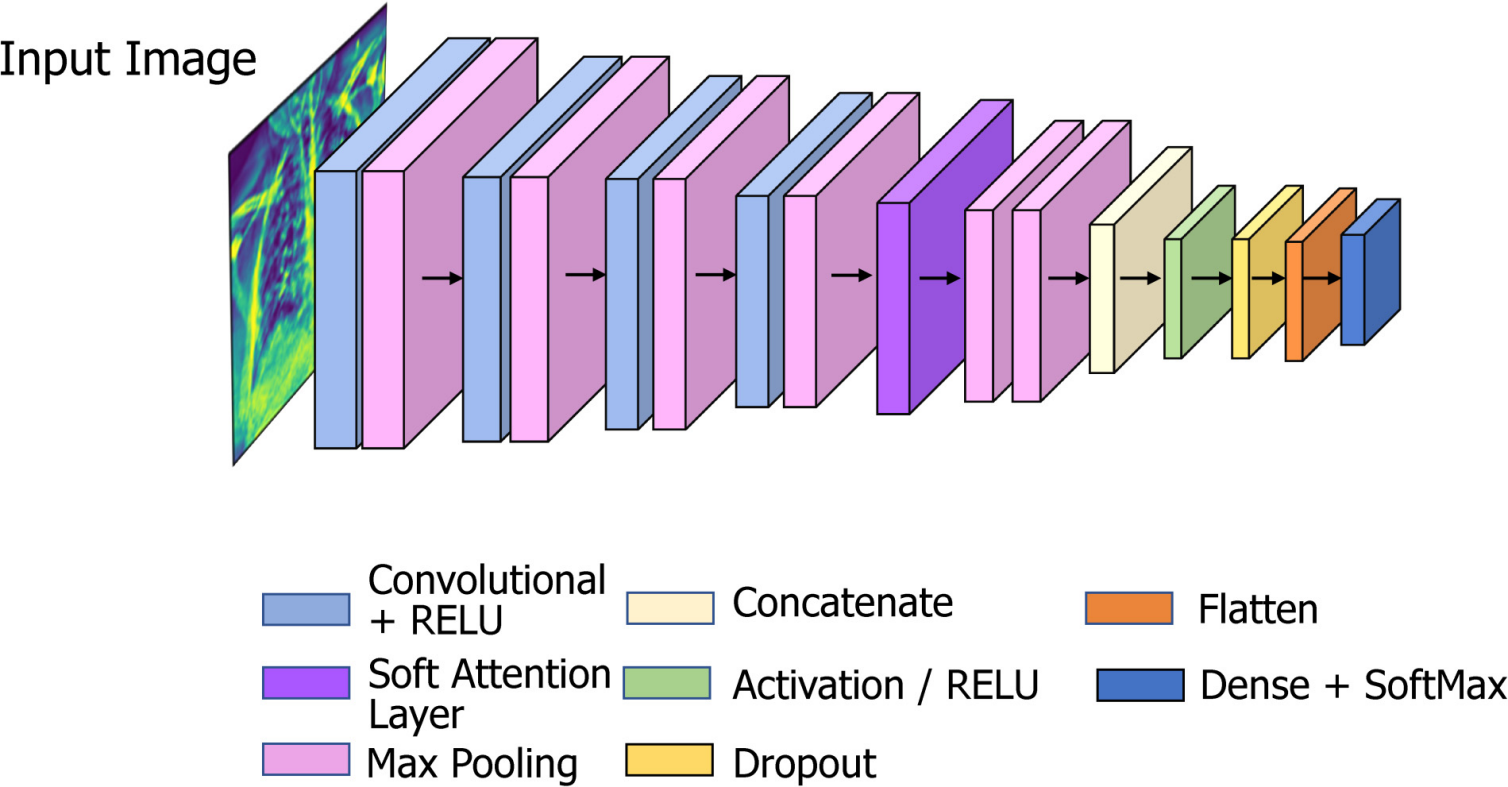
COV**-**X-net19 model architecture.

The architecture of the proposed network is shown below in Figure 8.

The proposed model has 16 layers, with 4 convolutional layers, 6 max-pooling layers, 1 soft attention layer, 1 dropout layer with a factor of 0.5, and a final dense layer for classification. To improve classification performance, soft attention technique is implemented into the proposed CNN model and this layer is added after concated convolution layer and max-pooling layer. The architecture of soft attention layer is described in Figure 7 and in Figure 8 the soft attention layer is highlighted by dark orange color. With the use of this soft attention-based methodology, the model is able to dynamically concentrate on pertinent image areas, improving classification accuracy. Thus, the addition of the soft attention layer was crucial in helping our proposed custom CNN model to perform better in terms of classification. Before the final classification dense layer, a flatten layer is added. The same 3  ×  3 kernel size is used for the convolution layers, and nonlinearity is introduced by including the ReLU activation function. The ReLU activation function applies the function *f*(*x*)  =  max (0, *x*) element-wise to the input, effectively thresholding negative values to zero and allowing the positive values to pass through, this makes the model more efficient by speeding up the training process and reducing the likelihood of vanishing gradients.^9^ The activation function for ReLU is given below in equation (3):

(3) $$F(x{)}_{\mathrm{ReLU}}=\max(0,x)$$

The max-pooling layer kernel size is 2  ×  2. As seen in the Figure 8, the input layer is attached to the first Conv2D layer, and the dimension of the input image is 224  ×  224  ×  3. Firstly, the Conv2D layer and max-pooling layer transform the dimension from 224  ×  224  ×  3 to 111  ×  111  ×  16. Then, a second Conv2D layer and a max-pooling layer transform the dimension from 111  ×  111  ×  16 to 54  ×  54  ×  32. The following process was repeated two more times and the dimension became 12 × 12 × 64. Then a soft attention layer is added to make sure the important parts of the images are the main emphasis. Then two max-pooling layers are added followed by a concatenation layer to concatenate where the output from the concatenation layer went through the Activation, Dropout, and flatten layers, respectively. Finally, it passes through the classification dense layer which is equipped with the SoftMax activation function. The SoftMax function is used for multiclassification models, and returns the probabilities of each class, with a high probability for the target class.^3^ The following is an expression for the SoftMax function given in equation (4):

(4) $$S_{i}=\frac{e^{x_{i}-\;x_{\max}}}{\sum_{j}^{\infty }\;e^{x_{j}-\;x_{\max}}}$$

### Transfer learning models

As previously said, we experimented with a total of eight models, including VGG16, VGG19, InceptionV3, MobileNetV1, MobileNetV2, DenseNet201, EfficientNetB1, EfficientNetB5 to compare the performance with our proposed CNN model. Transfer learning is a common deep-learning technique currently used in computer vision systems especially in the classification tasks using CXR images.^39^

#### VGG16

The VGG16 architecture consists of 16 layers, including 13 convolutional layers, 5 max-pooling layers, and 3 fully connected layers.^34^ The convolutional layers use small 3  ×  3 filters with a stride of 1 and a padding of 1, and the max-pooling layers use 2  ×  2 filters with a stride of 2. Convolutional and fully connected layers, that are highly connected in the model, allow for enhanced feature extraction and the usage of max-pooling (instead of average pooling).^2,40^ The network also uses a large number of filters in each convolutional layer, which further improves the quality of the learned features.

#### VGG19

VGG19 is a deep CNN architecture that is an extension of the VGG16 architecture. The VGG19 architecture consists of 19 layers, including 16 convolutional layers, 5 max-pooling layers, and 3 fully connected layers.^41^ The convolutional layers use small 3  ×  3 filters with a stride of 1 and a padding of 1, and the max-pooling layers use 2  ×  2 filters with a stride of 2. The main difference is that VGG19 has three additional convolutional layers compared to VGG16, which makes it a deeper network with more parameters.

#### InceptionV3

By changing previous Inception designs, InceptionV3 aims to reduce the necessary computational power. When compared to earlier Inception models, InceptionV3 contains a few significant improvements, such as label smoothing, factorized convolutional layers, and the use of an additional classifier to send label information down the network. By swapping out larger convolutions for smaller convolutions, the InceptionV3 model reduces training time.

#### MobileNetV1

In 2017, a new CNN architecture called MobileNet was introduced. The specialty of the model is that it is depth-wise and that its convolutions are placed depth-wise. Convolutions are applied to each channel rather than to the entire architecture all at once which reduces the cost of computations.^39^

#### MobileNetV2

Using depth-wise separable convolution as effective building blocks, MobileNetV2 is built on the principles of MobileNetV1. But in this revision, a new layer module known as the inverted residual with linear bottleneck is introduced. Achieving excellent performance with fewer resources is made possible by applying this compact and economical architecture.^42,43^

#### DenseNer201

The “Dense Block,” is the main component of DenseNet-201. Each layer in a dense block has several levels which receive input from all of the previous layers. The network maintains and improves the flow of information throughout the network by concatenating the feature maps from all previous levels. Transition layers, which carry out down sampling and cut down on the number of feature maps to regulate the model's complexity in DenseNet-201, connect the Dense Blocks.^44^ A batch normalization layer, a 1  ×  1 convolutional layer, and a 2  ×  2 average pooling layer make up these transition layers. With fewer feature maps, the transition layers guarantee that information flow is kept under control.

#### EfficientNetB1

By employing a compound scaling strategy, EfficientNetB1 achieves a balance between model performance and efficiency. It simultaneously and carefully scales the network's depth, breadth, and resolution. In order to identify the ideal values, a methodical grid search is used to determine the scaling coefficients.^45^ The “MBConv” block, which stands for Mobile Inverted Bottleneck Convolution, is the main component of EfficientNetB1. Depth-wise separable convolutions and bottleneck structures make up the MBConv block. By employing a 1  ×  1 convolutional layer (bottleneck) to cut down on the number of input channels before using a depth wise separable convolution, it effectively lowers the computational cost.

#### EfficientNetB5

The objective of EfficientNetB5 is to preserve computational efficiency while achieving a high level of accuracy. It adheres to the principles of compound scaling, which entails scaling the network's depth, width, and resolution all at once. There are seven stages in EfficientNetB5,^46^ each with a different number of MBConv blocks. In order to get the best values, a grid search is used to calculate the scaling coefficients for depth, width, and resolution. In general, EfficientNetB5 is wider and deeper than EfficientNetB1, making it a more potent and expressive model.

## Feature analysis

In this study, by identifying and extracting relevant features from image data, we aim to find out the features from the image, for which most images are being misclassified. Twenty-four clinically important features are extracted from the X-ray images which represent different perspectives of the lung area. The features include (i) area, (ii) perimeter area (PA) ratio, (iii) solidity, (iv) circularity, (v) equivalent diameter, (vi) convex area, (vii) extent, (viii) filled area, (ix) major axis length, (x) minor axis length, (xi) mean, (xii) standard deviation, (xiii) Shannon entropy, (xiv) Gray-Level Co-occurrence Matrix (GLCM) entropy, (xv) skewness, (xvi) kurtosis, (xvii) Local Binary Patterns (LBP) energy, (xviii) LBP entropy, (xix) Gabor energy, (xx) Gabor entropy, (xxi) contrast, (xxii) dissimilarity, (xxiii) energy, and (xxiv) correlation. Feature selection is a crucial part of finding out the optimal features. In previous studies, there are many state of art feature selection techniques have been implemented. As well as, the NB-GWOA approach, the genetic algorithm improves the use of the whale optimization algorithm to find the best solutions.^47^ Quantum-Behaved Multiverse Optimization^48^ views the features as particles in a multiverse, each of which stands for a subset of the features. Another feature selection method the Binary Ant Lion Optimizer is a binary version of the Ant Lion Optimizer algorithm that is used for optimization problems and^49^ for the selection of voice features. Moreover, to choose the ideal feature subset, the hybrid chaotic map and binary crow search optimization (CCSO) technique might be used.^50^ Another approach random forest feature ranking can be used to rank features according to their significance or contribution to the performance of the model, in addition to its predictive ability.^51^ MRMR^51^ is a feature selection method that looks for a subset of characteristics with a high level of relevance to the target variable while reducing redundancy among the chosen features. For analyzing the features, we have applied a statistical technique used to compare values between two or more groups called ANOVA test, random forest feature ranking, MRMR feature selection techniques.

### ANOVA test

The ANOVA test is used to analyze the variation within and between groups, and it can be used to test the null hypothesis that there is no significant difference between the means of groups. When conducting an ANOVA test, the data is first divided into groups or categories based on the independent variable. The test then measures the variation between the groups and the variation within the groups to determine whether the differences between the groups are statistically significant.^52^

## Result

In this section we discuss the findings, and the steps made for increasing accuracy, considering various methods for model evaluation and outcomes of the numerous ablation studies, to further assess the efficacy of the proposed X-net19 model. A description of the confusion matrix, accuracy loss curves, and performance evaluation matrix is also presented in this part.

### Evaluation matrix

To evaluate the model, we tested several matrices: precision, recall, F1-score, accuracy (ACC), sensitivity, and specificity. We generated a confusion matrix for the proposed model. The confusion matrix shows the ways in which a classification model is confused when it makes predictions.^10^ From the confusion matrix, we get the following outputs: true positive, indicates that the model correctly categorizes the positive class; true negative, indicates that the model correctly predicts the negative class; false positive (FP), indicates that the model incorrectly predicts the positive class; and false negative (FN), indicates that the model incorrectly predicts the negative class. AUC values have also frequently been calculated. In addition, for statistical analysis of the models, the FP rate, FN rate, false discovery rate, mean absolute error, and root mean squared error, are also calculated.

### Results of the ablation study

Five experiments are conducted as part of ablation study in order to enhance the performance of the proposed CNN model. As a more robust architecture with improved classification accuracy can be achieved by changing the components of CNN, ablation study is carried out for several components, including batch size, flatten layer, loss function, optimizer, and learning rate.

#### Ablation study 1: Changing the activation function

A neuron's activation status is determined by an activation function. By utilizing simpler mathematical procedures, it will determine whether or not the neuron's input to the network is significant during the prediction process.

In this case, we evaluate five different activation functions: hyperbolic tangent (tanh), Rectified Linear Unit (ReLU), Exponential Linear Unit (elu), softsign, softplus. It can be observed from Table 4 that the outcomes for these activation functions are 92.34%, **95.18%,** 93.42%, 88.05%, and 77.64% accordingly. The highest accuracy is obtained from ReLU activation function.

**Table 4.** Ablation study by changing activation function.

Case study	Activation	Result	Findings
1	tanh	92.34%	Identical accuracy
	**RelU**	**95**.**18%**	**Highest accuracy**
	elu	93.42%	Identical accuracy
	softsign	88.05%	Accuracy increased
	softplus	77.64%	Accuracy dropped

#### Ablation study 2: Ablation study by changing the pooling layers

The size of the extracted features is reduced by pooling layers. As a result, it reduces the quantity of network computation and the number of parameters that must be learned. Max-pooling and average pooling are the two types of pooling layers used in the experiments (Table 5).

**Table 5.** Ablation study by changing pooling layers.

Case study	Pooling layers	Result	Findings
2	**Max**	**95** **.** **42%**	**Highest accuracy**
	Average	84.16%	Lower accuracy

In Table 5, it can be seen that from two types of pooling layers we obtain the highest test accuracy of 95.42% using max-pooling layer.

#### Ablation study 3: Ablation study by changing the optimizer

Optimizers are techniques that modify the neural network's properties, such as its weights and learning rate, in order to minimize loss. In this study, experiments are conducted with four different optimizers: Adam, Nadam, Stochastic Gradient Descent, and Adamax.

From Table 6, it can be observed that while using optimizer Nadam the highest test accuracy of 95.38% is obtained. For additional ablation studies, the Nadam optimizer is chosen.

**Table 6.** Ablation study by changing optimizer function.

Case study	Optimizer	Result	Findings
3	Adam	93.48%	Lower accuracy
	**Nadam**	**95**.**38%**	**Highest accuracy**
	SGD	86.18%	Lower accuracy
	Adamax	94.17%	Near highest accuracy

SGD: Stochastic Gradient Descent.

#### Ablation study 4: Ablation study by changing the learning rate

We conducted a follow-up experiment using the Nadam optimizer by changing the learning rate (0.01, 0.006, 0.001, 0.0008). The results are shown in Table 7.

**Table 7.** Ablation study by changing learning rate.

Case study	Learning rate		Result	Findings
4	0.01		88.18%	Lower accuracy
	0.006		82.32%	Lower accuracy
	**0**.**001**		**95**.**38%**	**Highest accuracy**
	0.0008		93.05%	Near highest accuracy

In Table 7, the highest accuracy is obtained from 0.001, whereas the accuracy percentage decreases with the other learning rates. Therefore, the 0.001 learning rate is selected for further ablation research.

#### Ablation study 5: Ablation study by changing the loss function

A loss function evaluates how effectively the neural network models learn the training data by comparing the target and predicted output values. We demonstrate five loss functions: Binary crossentropy, categorical crossentropy, mean squared error, mean absolute error, and mean squared logarithmic error as shown in Table 8.

**Table 8.** Ablation study by changing loss function.

Case study	Activation	Result	Findings
5	Binary crossentropy	92.32%	Lower accuracy
	**Categorical crossentropy**	**95**.**53%**	**Highest accuracy**
	Mean squared error	91.44%	Lower accuracy
	Mean absolute error	89.35%	Lower accuracy
	Mean squared logarithmic error	15.32%	Lowest accuracy

The study shows that the highest accuracy is achieved from categorical crossentropy. Therefore, categorical crossentropy is selected.

### Performance analysis of best model

After analyzing the various components in the model, the configuration of the proposed model is finalized according to the best performance of each component. Table 9 shows the overview of the configuration.

**Table 9.** Configuration of the proposed model after ablation study.

Configuration	Value
Activation function	Relu
Epochs	100
Loss function	Categorical crossentropy
Batch size	32
Learning rate	0.001
Kernel size	3
Image size	224 × 224
Optimization function	Nadam
Weight decay	0.004
Poling layer kernel size	3
Pooling layer	Max-pooling

These hyperparameters are selected based on the results of our ablation studies and a grid search over a range of values. Our goal is to maximize model performance while minimizing overfitting and computational complexity. The results suggest that the chosen hyperparameters were effective for the specific classification task and dataset used in this study.

### Statistical analysis of the X-net19 model

The final X-Net19 model is created by doing ablation studies on the base model, significantly improving classification performance. This is accomplished by altering and configuring the model in different ways. Table 10 displays some evaluation criteria for the proposed X-net19 model, along with statistical analysis.

**Table 10.** The performance evaluation of the X-Net19 model using a number of matrices.

Measure	Value
Recall	93.80%
Specificity	98.27%
Precision	96.49%
F1 Score	95.28%
FPR	1.72%
FNR	6.19%
FDR	3.51%
NPV	98.76%

FDR: false discovery rate; FNR: false negative rate; FPR: false positive rate; NPV: negative predictive value.

**Figure 9.**
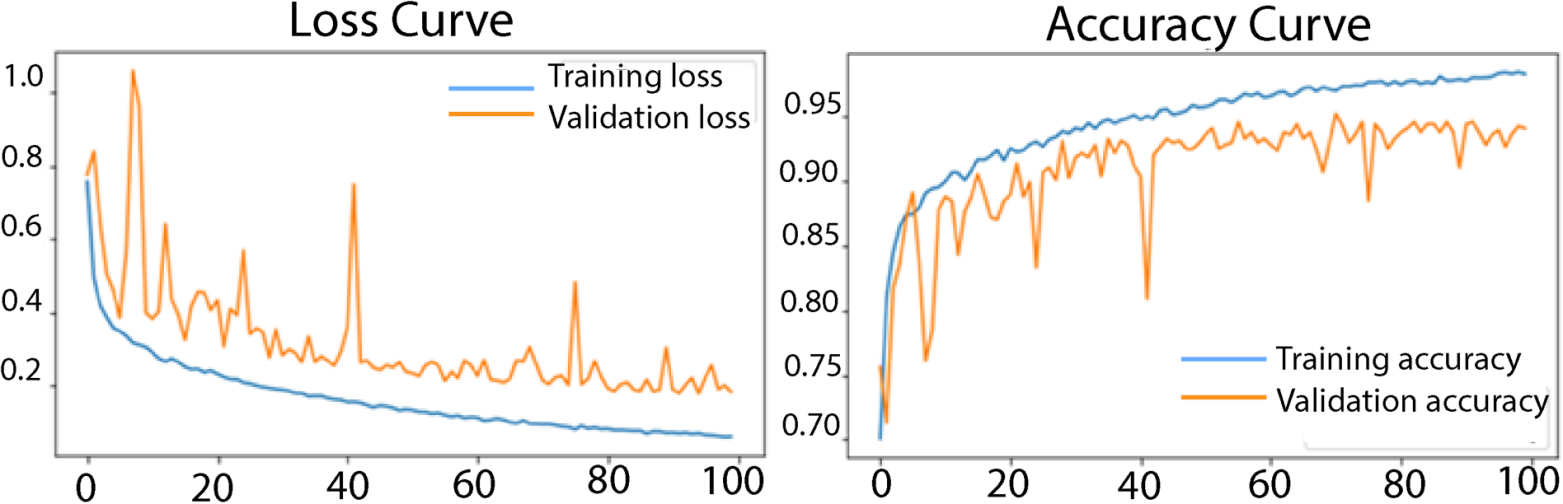
Loss curve and accuracy curve of the model after ablation study.

To further evaluate the performance of the X-net19 model, the loss and accuracy curves are plotted during training and validation. The accuracy and loss curves for the suggested model are shown in Figure 9.

As shown in Figure 9, both the training and validation accuracy increased over the course of training, while the loss decreased. However, the gap between the training and validation accuracy did not appear to increase substantially over time, suggesting no sign of overfitting. The loss and accuracy curves provide additional evidence that the proposed COV-X-net19 model is effective at classifying the target task. Moreover, we attained a training loss of 0.0619 and validation loss of 0.2141, conversely, for the Accuracy Curve we attained a training accuracy of 0.9826, validation accuracy of 0.9518 and testing accuracy of 0.9504.

**Figure 10.**
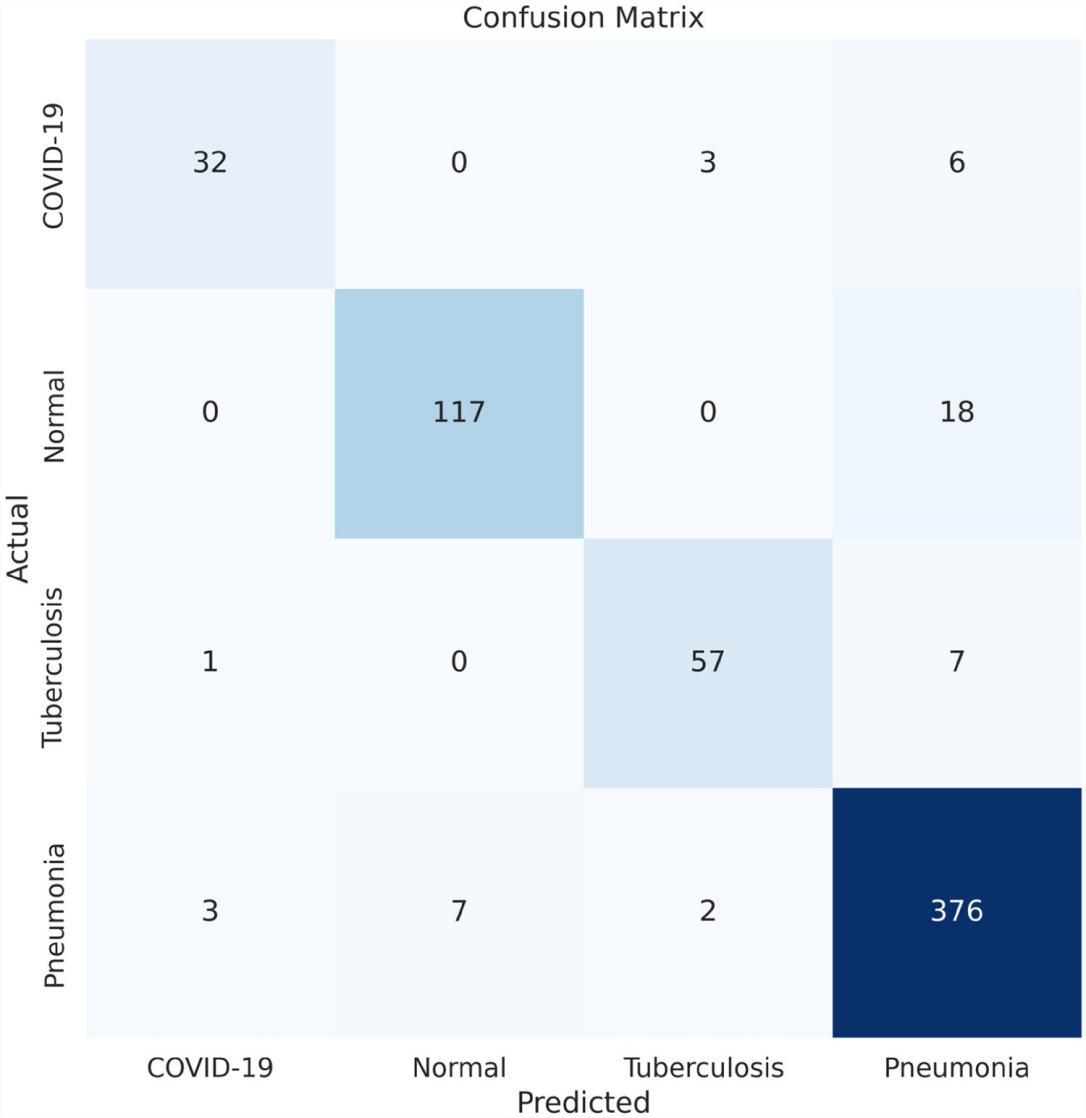
Confusion matrix after ablation study, x-axis represents the predicted value from the model and y-axis represents the actual value of each class.

The confusion matrix produced by the proposed model is displayed in Figure 10.

### Performance evaluation of the proposed model in comparison to transfer learning models

**Figure 11.**
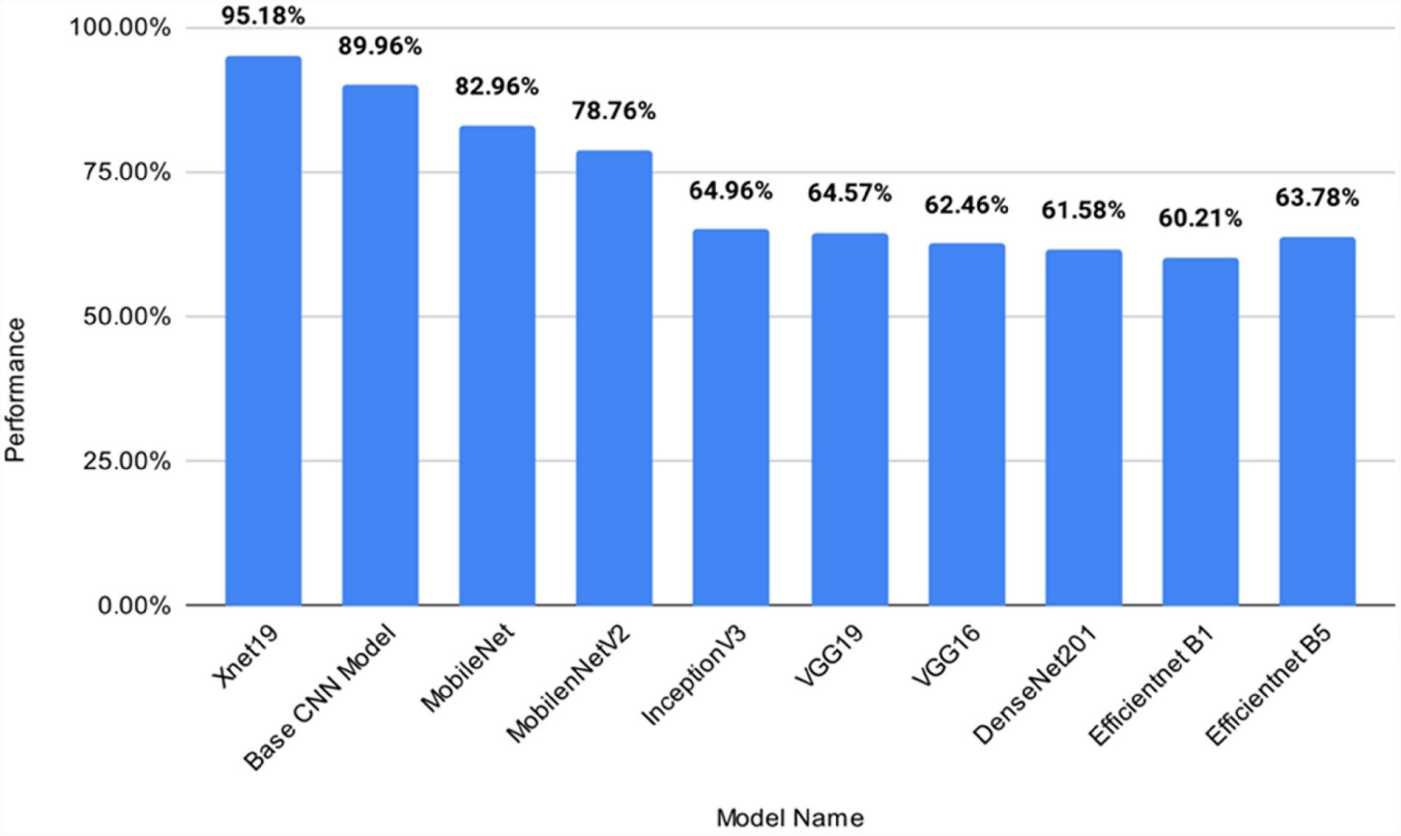
Performance analysis of the proposed model with transfer learning and base CNN model, where x-axis presents the model name and y-axis presents the performance(accuracy) of each model. CNN: convolutional neural network.

The performance comparison of transfer learning models with our proposed model. COV-X-net19 is showcased in Table 11 and Figure 11.

**Table 11.** Performance comparison of the proposed model with a transfer learning model.

Model	Number of params	Per epoch time (s)	Learning rate	Accuracy
VGG19	20,124,740	55–57	0.001	64.57%
VGG16	14,815,044	56–59	0.001	62.46%
MobileNetV2	2,508,868	161–170	0.001	78.76%
MobileNet	3,429,572	58–60	0.0007	82.96%
InceptionV3	22,007,588	57–60	0.001	64.96%
DenseNet201	213,312	134–140	0.001	61.58%
EfficientNetB1	2,418,349	20–30	0.001	60.21%
EfficientNetB5	3,429,572	20–30	0.001	63.78%
X-net19 (Customize Base Model)	1,579,860	52–54	0.001	95.18%

Based on Table 11 and Figure 11 comparing the proposed model with various transfer learning models, it is observed that the accuracy of these models ranges from 60.21% to 82.96%, while the base CNN model achieves an accuracy of 89.96%. The proposed X-net19 model outperforms all the other models with an accuracy of 95.18%, which is significantly higher than any other model. Therefore, it can be concluded that the proposed X-net19 model is superior to the other transfer learning models, and the base CNN model, in terms of accuracy.

### Comparison of accuracy of the proposed model changing image quality and image number

**Figure 12.**
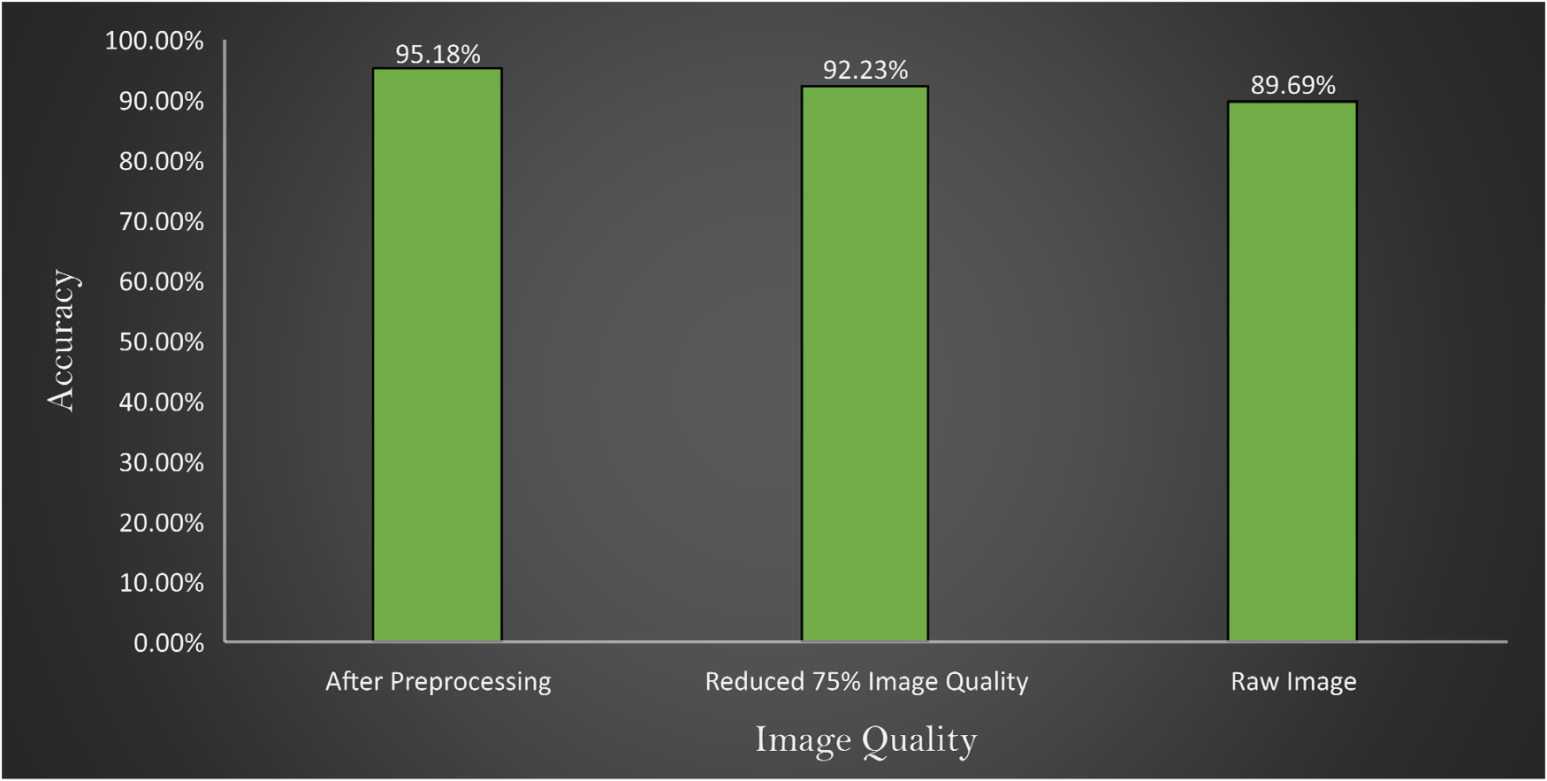
Comparison of accuracy with images after preprocessing, reducing 75% image quality of preprocessed image and raw image represented in x-axis where y-axis represents the accuracy obtains by the experiments.

In Figure 12, it is observed that the model provides an accuracy of 89.69% with a raw image, 95.18% with preprocessed image and 92.23% with the quality reduced image. From these experiments, two major findings can be stated:

1. When the performance is compared between preprocessed and raw images, there is a difference, indicating that the preprocessed image processing approaches are beneficial.
2. Across all the experiments an accuracy above 89% is achieved, which further validates the robustness of the proposed model.

Three experiments are conducted where at first the model is tested with raw images, then preprocessed image, and finally 75% quality reduced image. The outcome is illustrated in Figure 12.

### Robustness assessment

**Figure 13.**
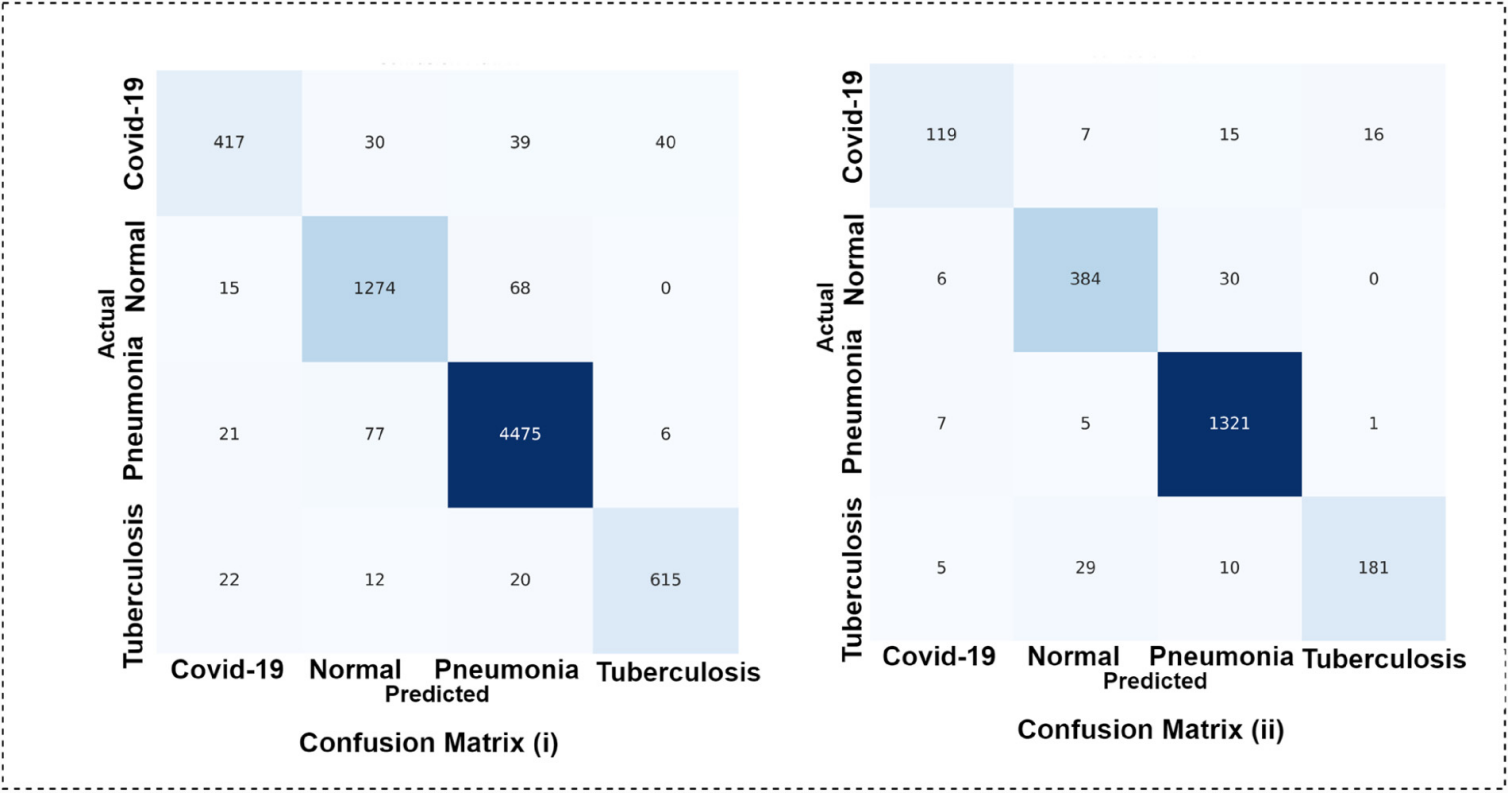
Confusion matrix (i) splitting ratio: 70%(train), 20%(test), 10%(validation) and confusion matrix (ii) splitting ratio: 60%(train), 30%(test), 10%(validation).

In order to assess the robustness of the proposed model even further, the model is trained twice with more images. The dataset is split using ratio of 70:10:20 and 60:10:30 for train, validation and test set, respectively. While splitting the dataset using a ratio of 70:10:20, 4995 images are found in train set, 714 in validation set, and 1427 in test set. For the splitting ratio of 60:20:10, 4281, 713 and 2141 images are found in train, validation and test set, respectively. Figure 13 showcases the confusion matrix for the two splitting scenarios.

From Figure 13, confusion matrix (i) shows that the model is able to obtain a test accuracy of 95.18% for 20% training images and confusion matrix; and (ii) shows that the model obtains test accuracy of 95.04% on the test set of 30%. Moreover, in class-based performance, the model is robust enough to predict all the classes with no bias. This experiment further validates the performance consistency and robustness of the proposed architecture.

### Analysis of misclassification through feature testing

The study applies ANOVA testing to find out the reason for misclassified images based on specific features including area, PA ratio, solidity, circularity, equivalent diameter, convex area, extent, filled area, major axis length, minor axis length, mean, standard deviation; and Shanon antropy, GLCM entropy, Skewness, Kurtosis, LBP energy, LBP entropy, Gabor energy, Gabor entropy, Contrast, Dissimilarity, Energy, Correlation.

Before extracting the features, the images are first segmented and then the lung regions are extracted. Anter et al.^53^ proposed a novel approach to enhance the fuzzy c-means (FCM) clustering technique for autonomous localization and segmentation of liver and hepatic lesions from CT scans. Our proposed algorithm involving segmenting the lung regions is stated in Algorithm 1.

**Algorithm 1.** This algorithm shows the process of extracting the ROI from the CXR images

1:	START
2:	Read image
3:	Convert image to grayscale
4:	Apply Gaussian blur to reduce noise
5:	Apply thresholding to segment the image
6:	Find contours in the segmented image
7:	Compute contour sizes for all contours
8:	Find the largest contour
9:	Extract shape features from the largest contour
10:	Display or use the extracted shape features as needed
11:	END

In this process, after reading an image, it is converted into grayscale format. Gaussian blur is applied to reduce noise. Using a thresholding approach, the picture is then divided into the foreground and background areas. The segmented image has contours, which are continuous curves that depict object boundaries. The size of all the contours is computed to find the two largest contours. Finally, from the largest two contours (lung regions) the abovementioned handcrafted features are extracted. The extracted features from CXR images described according its description shown individually in Table 12.

**Table 12.** Extracted features from CXR images with feature description.

No.	Feature name	Description
1	Area	Area of the desired region^54^
2	Perimeter area ratio	Perimeter area ratio determines the horizontal to vertical pixel ratio of an image^54^
3	Solidity	Solidarity is the ratio of the contour area and the smallest convex haul which covers the area^54^
4	Circularity	The circularity feature is utilized to determine the tumor's degree^54^
5	Equivalent Diameter	The equivalent diameter indicates the diameter of a circle with the same ROI surface area^54^
6	Convex area	The equivalent diameter indicates the diameter of a circle with the same ROI surface area^55^
7	Extent	The area of the segmented object is divided by the area of its convex hull denoted as the extent^55^
8	Filled area	The filled area is the interpolated pixel value that covers all the ROI areas^55^
9	Major axis length	Major axis length is the measurement of the pixel distance between the major axis endpoints of the object area^55^
10	Minor axis length	Minor axis length is the lowest length of the targeted pixel area^55^
11	Mean	Mean is the average pixel intensity^55^
12	Standard deviation	The standard deviation refers to the measurement of the variation of image gray-level intensities^54^
13	Shannon entropy	The average amount of information contained in the area is estimated using the Shannon entropy^55^
14	GLCM entropy	GLCM entropy calculates the texture feature contents of the segmented object^55^
15	Skewness	The skewness is a measurement used to assess the symmetry or asymmetry data distribution in the area^55^
16	Kurtosis	The kurtosis statistic determines whether the tails of a normal distribution of the ROI area are heavy or light1^55^
17	LBP energy	LBP energy is a texture primitive descriptor of LBP.^56^
18	LBP entropy	LBP entropy is a texture descriptor that combines the concept of LBP with entropy calculation^56^
19	Gabor energy	Gabor Energy means convoluting an image with a set of Gabor filters. It's a textual feature of GLCM^55^
20	Gabor entropy	Gabor entropy is a texture descriptor that combines the Gabor filter response with entropy calculation^55^
21	Contrast	Contrast is a texture feature that measures the difference in intensity between neighboring pixels within a texture region^57^
22	Dissimilarity	Dissimilarity is a texture feature that calculates the average absolute difference in intensity between neighboring pixels within a texture region^57^
23	Energy	Energy (also known as angular second moment) is a texture feature that measures the uniformity or homogeneity of the texture pattern^57^
24	Correlation	Correlation is a texture feature that measures the linear dependency between neighboring pixel intensities within a texture region^\^

CXR: chest X-ray; GLCM: Gray-Level Co-occurrence Matrix; LBP: Local Binary Patterns; ROI: region of interest.

An ANOVA test is first performed on classified images and then repeated on misclassified images. In the ANOVA test, the higher the F-value, the larger difference among the groups. The experimental results of the ANOVA test are shown in Table 13.

**Table 13.** ANOVA test between four classes with corresponding F-value and P-value.

No	Disease	Feature	F-value (classified)	F-value (misclassified)
1.	COVID-19, tuberculosis, normal, pneumonia	Area	70.404	5.170
2.	COVID-19, tuberculosis, normal, pneumonia	PA ratio	1417.586	1.314
3.	COVID-19, tuberculosis, normal, pneumonia	Solidity	157.343	7.599
4.	COVID-19, tuberculosis, normal, pneumonia	Circularity	182.455	0.024
5.	COVID-19, tuberculosis, normal, pneumonia	Equivalent Diameter	70.209	5.176
6.	COVID-19, tuberculosis, normal, pneumonia	Convex area	369.199	5.091
7.	COVID-19, tuberculosis, normal, pneumonia	Extent	259.134	0.714
8.	COVID-19, tuberculosis, normal, pneumonia	Filled area	180.509	0.875
9.	COVID-19, tuberculosis, normal, pneumonia	Major axis length	444.809	30.992
10.	COVID-19, tuberculosis, normal, pneumonia	Minor axis length	88.657	0.564
11.	COVID-19, tuberculosis, normal, pneumonia	Mean	84.146	0.002
12.	COVID-19, tuberculosis, normal, pneumonia	Standard deviation	139.354	0.909
13.	COVID-19, tuberculosis, normal, pneumonia	Shannon_Entropy	20.598	0.001
14.	COVID-19, tuberculosis, normal, pneumonia	GLCM entropy	577.706	0.073
15.	COVID-19, tuberculosis, normal, pneumonia	Skewness	79.225	2.326
16.	COVID-19, tuberculosis, normal, pneumonia	kurtosis	43.426	15.807
17.	COVID-19, tuberculosis, normal, pneumonia	LBP energy	295.880	0.390
18.	COVID-19, tuberculosis, normal, pneumonia	LBP entropy	351.455	0.107
19.	COVID-19, tuberculosis, normal, pneumonia	Gabor energy	9.360	0.070
20.	COVID-19, tuberculosis, normal, pneumonia	Gabor entropy	10.063	0.030
21.	COVID-19, tuberculosis, normal, pneumonia	Contrast	78.385	0.0425
22.	COVID-19, tuberculosis, normal, pneumonia	Dissimilarity	83.254	0.0385
23.	COVID-19, tuberculosis, normal, pneumonia	Energy	473.517	0.0134
24	COVID-19, tuberculosis, normal, pneumonia	Correlation	71.419	0.317

ANOVA: analysis of variance; LBP: Local Binary Patterns; PA: perimeter area.

It is observed from Table 13 that, in terms of correct classification, the F-value is higher across all the features (around 9-1417). However, for the misclassified images, the F-values are found to be significantly lower (around 0–30). This further demonstrates that in the correctly classified images, the imaging feature differences are relatively higher, whereas in misclassified images; the images are quite similar in terms of features.

**Figure 14.**
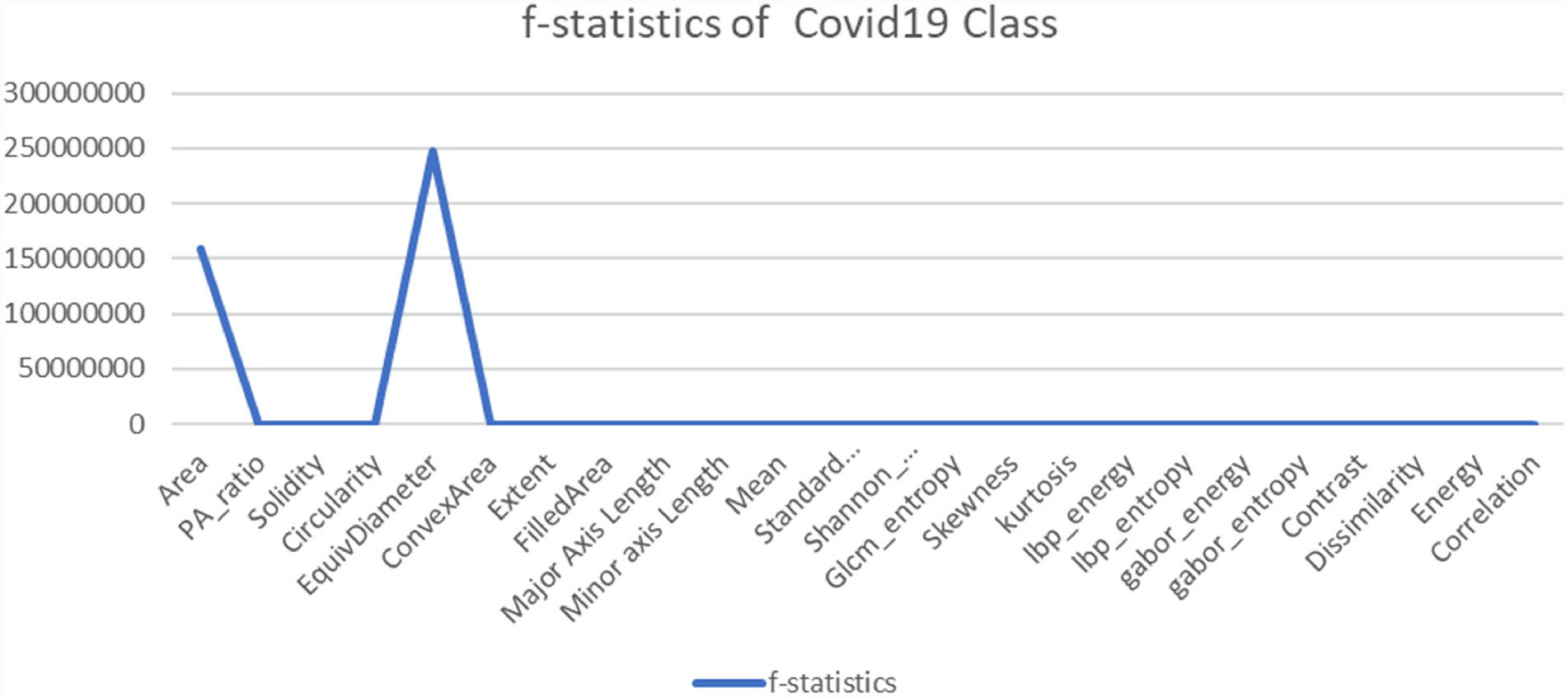
ANOVA test results for COVID-19 class between classified and misclassified images. ANOVA: analysis of variance.

**Figure 15.**
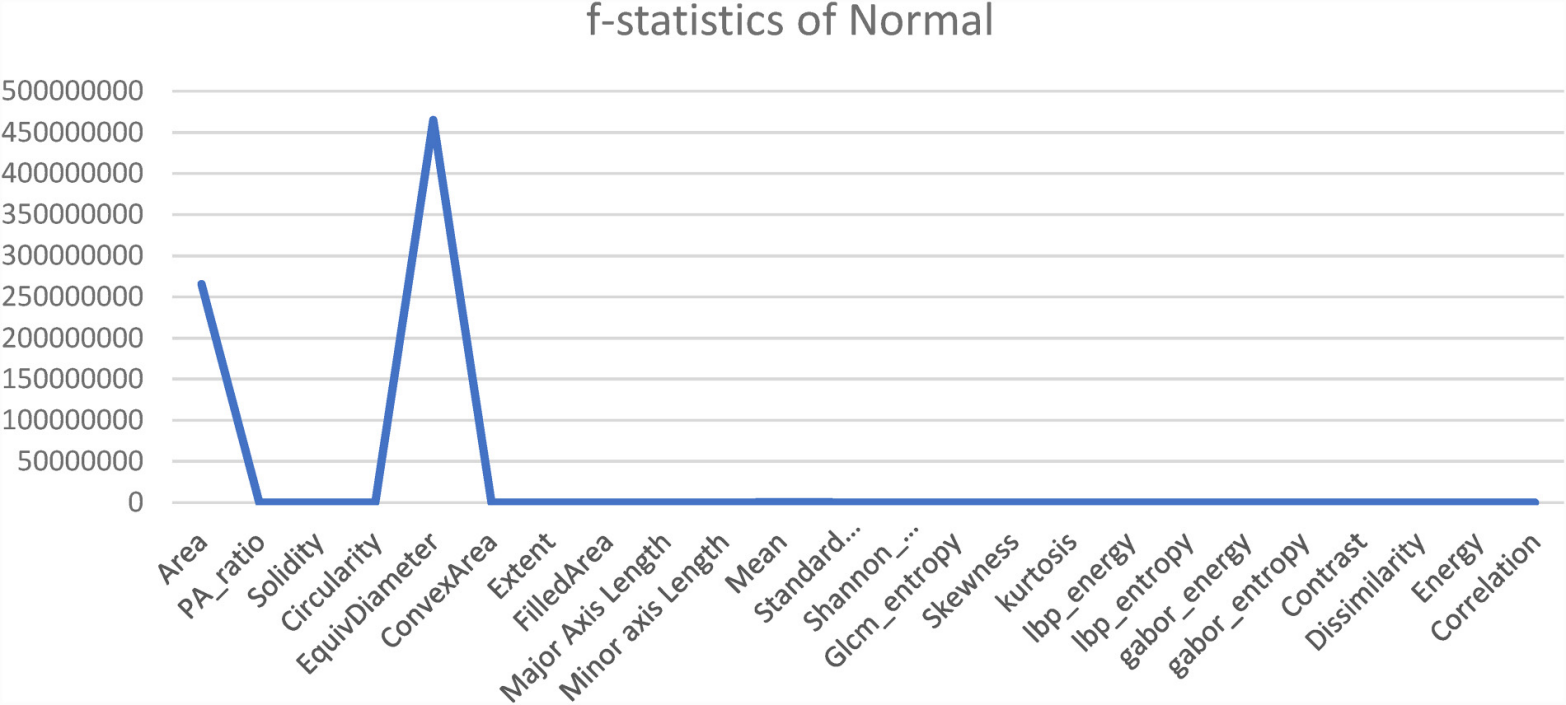
ANOVA test results for normal class between classified and misclassified images. ANOVA: analysis of variance.

**Figure 16.**
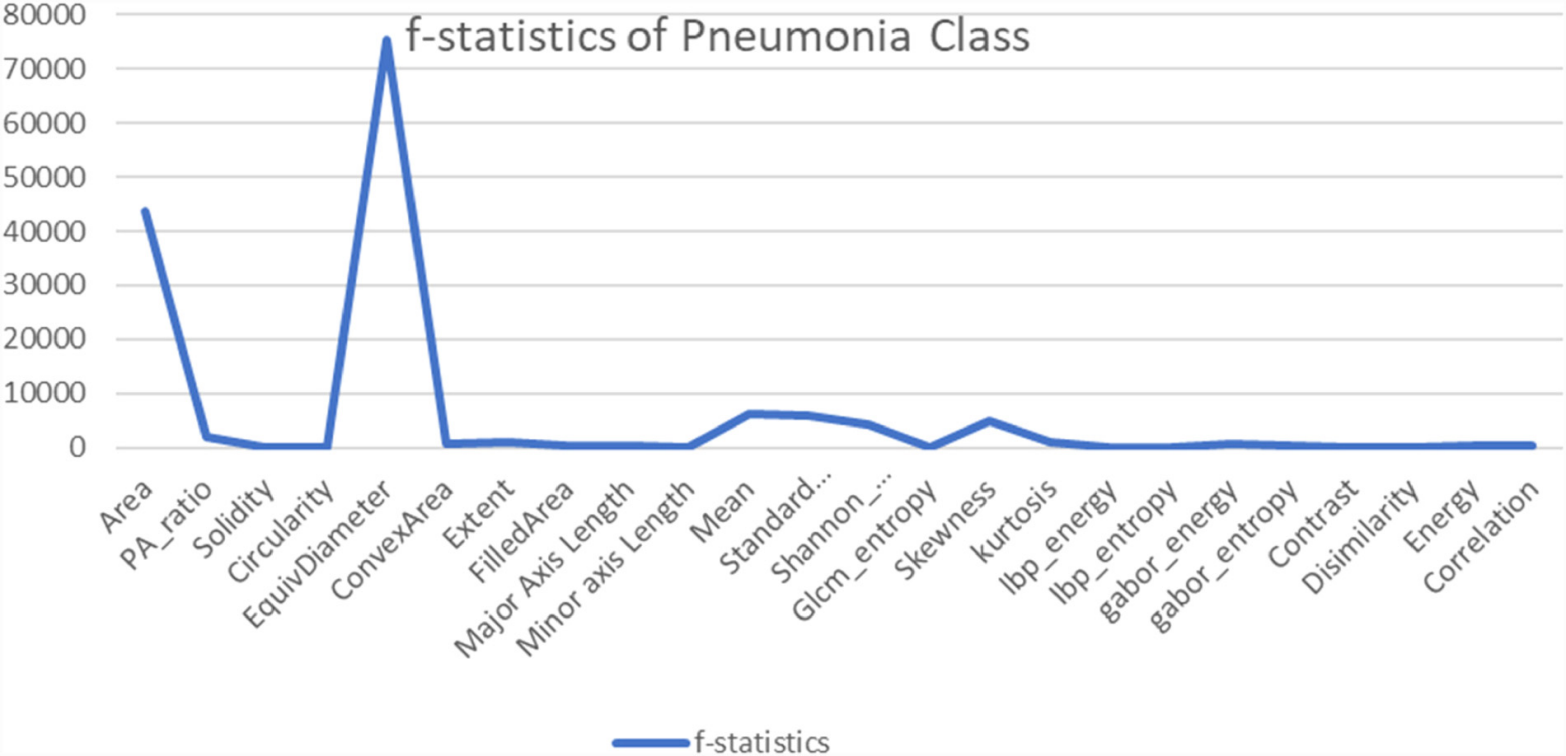
ANOVA test results for pneumonia class between classified and misclassified image. ANOVA: analysis of variance.

**Figure 17.**
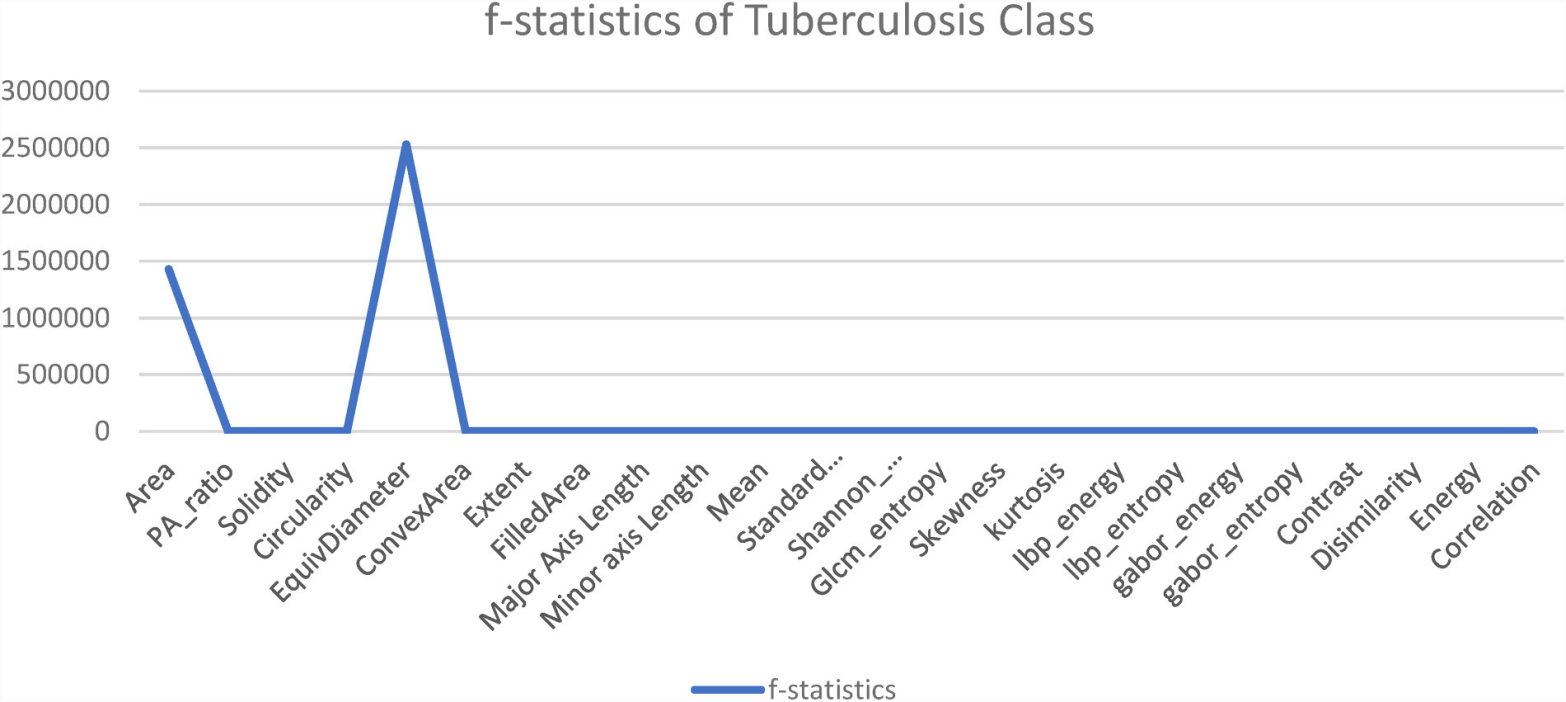
ANOVA test results for tuberculosis class between classified and misclassified image. ANOVA: analysis of variance.

Another statistical analysis is conducted to compare the F-value between classified images and misclassified images of each class. The ANOVA test is applied to the classified image set and again to the misclassified images for the particular class of every feature. A higher F-value means this feature has a huge difference compared to a classified image feature and has no correlation with the features for which an image can be classified. In Figures 14–17 the feature “Equivalent Diameter” conveys the F-value 247,090,263.43, 2,529,159.382, 465,088,529.26, and 75,496.73 particularly. In this analysis it is found that, “Equivalent Diameter” represents the highest F-value and indication of misclassification.

**Figure 18.**
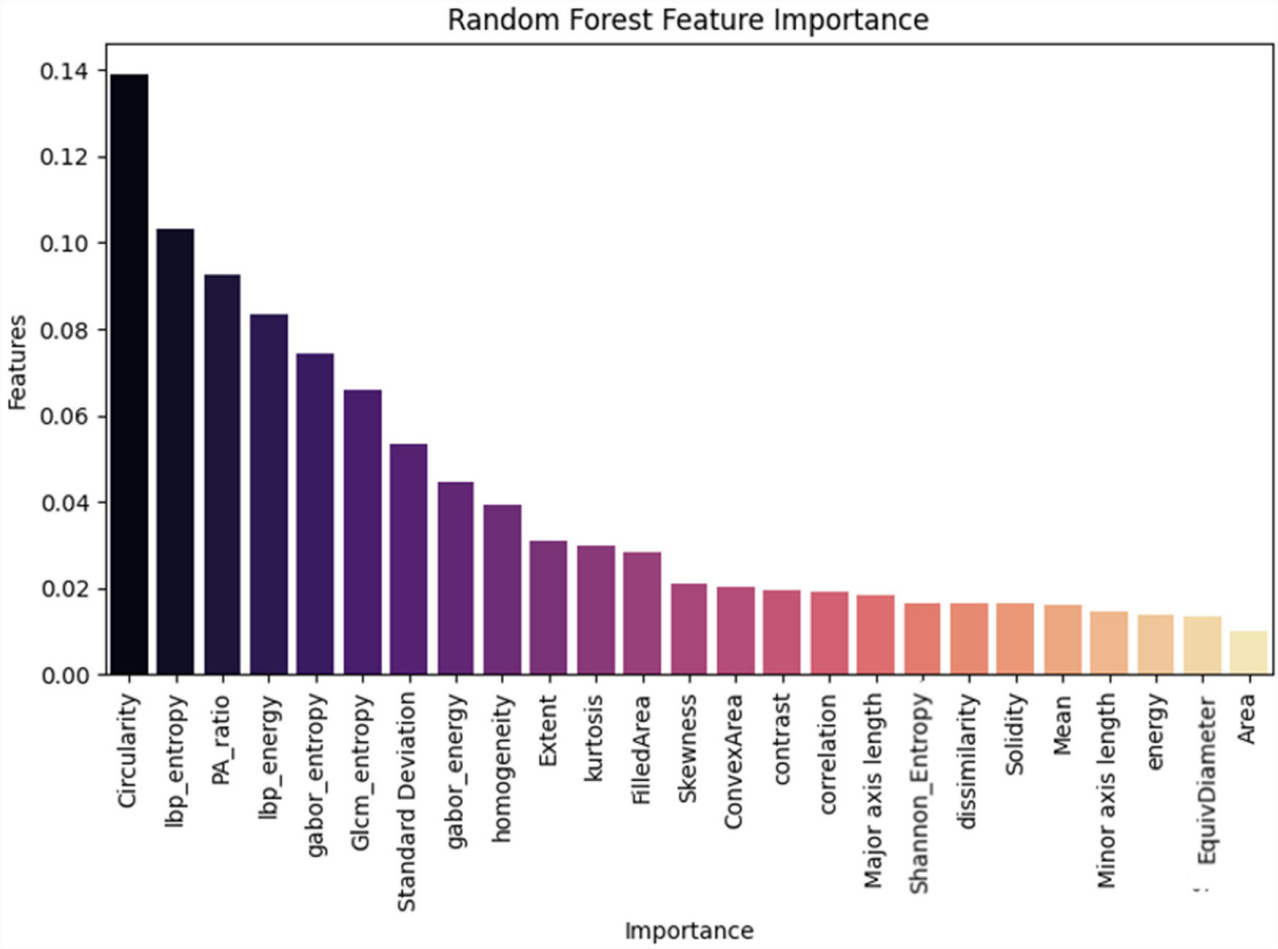
Random forest feature importance between all feature.

In order to determine the value of various characteristics throughout our investigation, we used the random forest feature importance approach. The findings showed that “area” and “equivalent diameter,” two specific factors, received the lowest ranks in terms of relevance. Remarkably, the results of an additional statistical test ANOVA confirmed similar conclusions. The results are shown in Figures 14–18.

**Figure 19.**
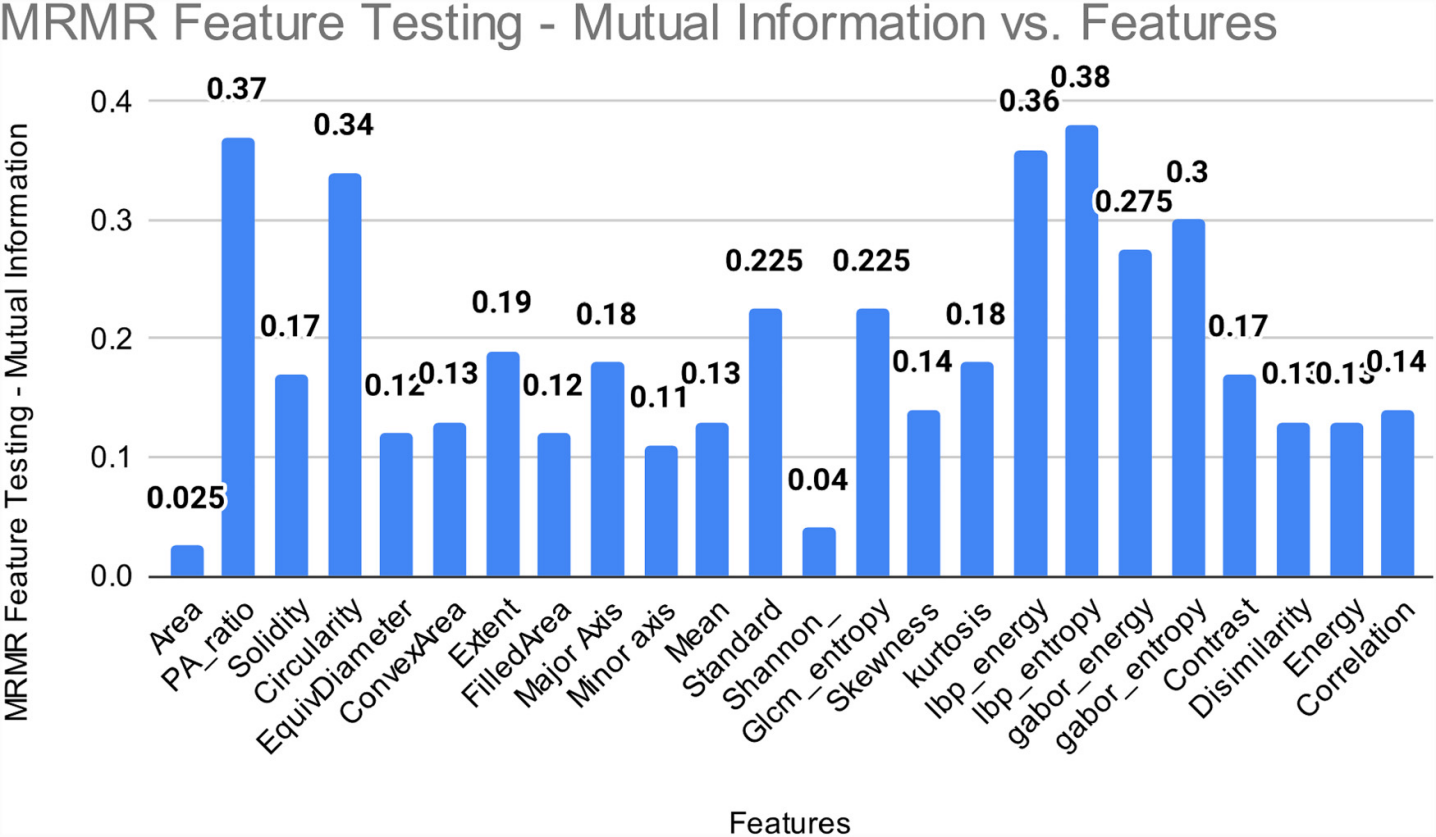
MRMR feature testing. MRMR: Minimum Redundancy Maximum Relevance.

Moreover, we have performed MRMR feature testing utilizing the mutual information metrics in addition to the previous research. By eliminating redundancy among the chosen characteristics, the MRMR method seeks to uncover features that demonstrate high relevance to the target variable. It's important to point out that the feature “area” had a relatively low mutual information value throughout our MRMR feature testing. This suggests that the “area” feature may only have partial knowledge of or limited ability to forecast the target variable. Additionally, it suggests that the misclassification or errors in prediction may be related to the “area” feature when taking consideration of the outcomes from other testing methodologies. The results are shown in Figure 19.

## Limitations

The proposed methodology used a robust model with impactful image preprocessing and get a good accuracy, though there are limitations too. Working with real data is always complex task both in preprocessing and model training. For unavailability of private data this work can’t be tested on private data for finding out the more robustness of the model. Additionally, optimization of the proposed model can be another limitation of this work. This model could be more optimized by implementing the scratch of the model's different layers for getting more accuracy. However, in spite of this limitation our proposed methodology performs well in the particular dataset and able to identify the features for which the model misinterpreted the images.

## Future work

In future attempts, the focus will be given to expanding the dataset in order to improve the robustness and expansion abilities of our model. A larger dataset gives the model access to a wider variety of data, which enables it to discover more typical patterns and characteristics. Moreover, the robustness of the proposed approaches can be evaluated further using real-world medical data. The performance of the proposed model can be validated in real-world scenarios and its practical application can be determined by integrating actual medical images gathered from clinical settings or medical databases. Real data can come with particular difficulties and complexity. Our future work might concentrate on finding clinical information using handcrafted features in addition to the model's capacity to learn features automatically. We strive to build pertinent characteristics that capture particular aspects of medical disorders under research by utilizing domain-specific information and expert perspectives. This strategy will improve the predictions of the model’s interpretability and domain-specific comprehension, aiding clinical decision making and diagnosis.

## Conclusion

In this study, an attention mechanism-based CNN model is proposed to diagnose three specific diseases: COVID-19, pneumonia, and tuberculosis, with 95.18% accuracy. Several image processing techniques are applied to preprocess CXR images. A soft attention mechanism is used to increase model accuracy. For the evaluation of the model, an ablation study is carried out with the proposed model. Pretrained transfer learning models are applied for comparison with the proposed model. Furthermore, the reasons for misclassified images are analyzed through statistical evaluation. By undertaking feature analysis, 24 features are extracted from both the classified image and the misclassified image. Then a statistical analysis is performed where the ANOVA test is applied. The proposed approach is a novel method for early diagnosis of pulmonary disease and in identifying the reasons for misclassifying images which can significantly assist medical specialists in achieving accurate pulmonary disease classification and diagnosis.
